# Comparing Laparoscopic Elective Sigmoid Resection With Conservative Treatment in Improving Quality of Life of Patients With Diverticulitis: The Laparoscopic Elective Sigmoid Resection Following Diverticulitis (LASER) Randomized Clinical Trial

**DOI:** 10.1001/jamasurg.2020.5151

**Published:** 2020-11-18

**Authors:** Alexandre Santos, Panu Mentula, Tarja Pinta, Shamel Ismail, Tero Rautio, Risto Juusela, Aleksi Lähdesmäki, Tom Scheinin, Ville Sallinen

**Affiliations:** 1 Gastroenterological Surgery, University of Helsinki, Helsinki University Hospital, Helsinki, Finland; 2 Department of Surgery, Seinäjoki Central Hospital, Seinäjoki, Finland; 3 Department of Surgery, Medical Research Center, Oulu University Hospital, Oulu, Finland; 4 Department of Surgery, Vaasa Central Hospital, Vaasa, Finland; 5 Department of Surgery, Hyvinkää Hospital, Hyvinkää, Finland; 6 Transplantation and Liver Surgery, University of Helsinki, Helsinki University Hospital, Helsinki, Finland

## Abstract

**Importance:**

Diverticulitis has a tendency to recur and affect quality of life.

**Objective:**

To assess whether sigmoid resection is superior to conservative treatment in improving quality of life of patients with recurrent, complicated, or persistent painful diverticulitis.

**Design, Setting, and Participants:**

This open-label randomized clinical trial assessed for eligibility 128 patients with recurrent, complicated, or persistent painful diverticulitis in 6 Finnish hospitals from September 29, 2014, to October 10, 2018. Exclusion criteria included age younger than 18 years or older than 75 years; lack of (virtual) colonoscopy or sigmoidoscopy data within 2 years, or presence of cancer, contraindication to laparoscopy, or fistula. Outcomes were assessed using intention-to-treat analysis. A prespecified interim analysis was undertaken when 66 patients had been randomized and their 6-month follow-up was assessable. Data were analyzed from June 2018 to May 2020.

**Interventions:**

Laparoscopic sigmoid resection or conservative treatment.

**Main Outcomes and Measures:**

The primary outcome was difference in Gastrointestinal Quality of Life Index (GIQLI) score between randomization and 6 months.

**Results:**

Of 128 patients assessed for eligibility, 90 were randomized (28 male [31%]; mean [SD] age, 54.11 [11.9] years; 62 female [69%]; mean [SD] age, 57.13 [7.6] years). A total of 72 patients were included in analyses for the primary outcome (37 in the surgery group and 35 in the conservative treatment group), and 85 were included in analyses for clinical outcomes (41 in the surgery group and 44 in the conservative treatment group). The difference between GIQLI score at randomization and 6 months was a mean of 11.96 points higher in the surgery group than in the conservative treatment group (mean [SD] of 11.76 [15.89] points vs −0.2 [19.07] points; difference, 11.96; 95% CI, 3.72-20.19; *P* = .005). Four patients (10%) in the surgery group and no patients in the conservative treatment group experienced major complications (Clavien-Dindo grade III or higher). There were 2 patients (5%) in the surgery group and 12 patients (31%) in the conservative treatment group who had new episodes of diverticulitis within 6 months.

**Conclusions and Relevance:**

In this randomized clinical trial, elective laparoscopic sigmoid resection improved quality of life in patients with recurrent, complicated, or persistent painful diverticulitis but carried a 10% risk of major complications.

**Trial Registration:**

ClinicalTrials.gov Identifier: NCT02174926.

## Key Points

**Question:**

Does elective sigmoid resection improve the quality of life in patients with recurrent diverticulitis, complicated diverticulitis, and/or chronic pain after an episode of diverticulitis?

**Findings:**

In this randomized clinical trial that included 85 adults, the Gastrointestinal Quality of Life Index (GIQLI) score improved 11.8 points in patients randomized to sigmoid resection and 0.2 points in patients randomized to conservative treatment between baseline and 6 months, a statistically and clinically significant difference. Among 41 patients randomized to sigmoid resection, 4 (10%) experienced major complications.

**Meaning:**

Sigmoid resection improved quality of life in patients with recurrent, complicated, or persistent painful diverticulitis but was associated with a small but significant risk of major complications.

## Teaser

This randomized clinical trial assesses whether sigmoid resection is superior to conservative treatment in improving quality of life of patients with recurrent, complicated, or persistent painful diverticulitis.

## Introduction

Diverticular disease is a common disorder ranging from 10% in those younger than 40 years to between 50% and 70% in those older than 80 years.^1^ The most frequent complication, acute diverticulitis, can range from mild uncomplicated diverticulitis, which requires no antibiotics,^2,3,4^ to life-threatening peritonitis.^5^

As the vast majority of episodes of acute diverticulitis are uncomplicated,^5,6^ the bowel segment affected by the diverticular disease is typically left intact and is prone to develop recurrent diverticulitis. Historically, even a single recurrence in young patients or a second recurrence in elderly patients often led to elective sigmoid resection to prevent recurrence.^7^ However, the first episode of acute diverticulitis is usually the most severe, and recurrences rarely require emergency surgery.^8,9^ Therefore, the treatment recommendation has shifted to a more conservative and tailored approach taking into account the complaints and risks associated with elective sigmoid resection, not the number of recurrences.^6,10,11^ These guidelines are based on the ability of elective sigmoid resection to prevent dangerous complicated recurrences, which are, in fact, rare.^12^ Guidelines also recommend elective sigmoid resection after an abscess has been treated conservatively, but some have argued that elective sigmoid resection could be safely omitted after complicated diverticulitis.^13^ However, recurrent diverticulitis reduces patients’ quality of life (QOL), whereas QOL could be improved by elective sigmoid resection.^14,15,16^ The evidence regarding the beneficial effects of elective sigmoid resection has been mostly based on low-quality, uncontrolled retrospective series.^17^ To our knowledge, the first and only randomized clinical trial comparing conservative treatment with elective sigmoid resection in patients with recurrent diverticulitis or persistent complaints after an episode of diverticulitis was published in 2016 (DIRECT trial).^18,19^

To compensate for the lack of evidence, the Laparoscopic Elective Sigmoid Resection Following Diverticulitis (LASER) trial was commenced. Our hypothesis was that elective sigmoid resection would improve QOL of patients who have recurrent, complicated, or persistent diverticulitis.

## Methods

### Study Design

The LASER trial was a multicenter, prospective, parallel, open-label randomized clinical trial comparing elective laparoscopic sigmoid resection with conservative treatment in patients with either recurrent, complicated, or persistent painful diverticulitis. The trial was carried out in 2 academic university hospitals (Helsinki and Oulu university hospitals) and 3 community hospitals (Seinäjoki Central, Vaasa Central, and Hyvinkää hospitals) in Finland. This study followed the Consolidated Standards of Reporting Trials (CONSORT) reporting guideline. The trial protocol was approved by the ethical committee of the Helsinki University Hospital and institutional review boards at each site and can be found in Supplement 1. The trial was registered in ClinicalTrials.gov before commencement. A new study site (Hyvinkää Central Hospital) was added to the protocol, but no other changes were made to the protocol after trial commencement. All patients gave written informed consent before randomization.

### Participants

Patients were eligible for inclusion if they had 3 or more episodes of left colon diverticulitis within a 2-year period with at least 1 episode verified using computed tomography (CT); 1 or more episodes of conservatively treated complicated left colonic diverticulitis; and/or prolonged pain or disturbance in bowel habits more than 3 months after an episode of CT-verified acute left colonic diverticulitis. Complicated diverticulitis was defined as diverticulitis with fistula, stricture, abscess, or free air in the abdominal cavity verified by CT. Diverticulitis with pericolic air only was not considered as complicated diverticulitis.^5^ Patients who had multiple morbidities that prevented elective surgery; contraindication to laparoscopy; colonic stricture; fistula (eg, colocutaneous, colovaginal, colovesical); active malignancy; earlier resection of sigmoid colon or rectum; acute diverticulitis that had not settled (eg, elevated inflammatory markers, fever); had not had a colonoscopy, sigmoidoscopy, or virtual colonoscopy performed within 2 years; were younger than 18 years or older than 75 years; were pregnant; or were unable to answer the health survey (eg, because of dementia or a psychiatric condition) were excluded from the trial.

### Randomization

Patients were randomly allocated (1:1) to receive either elective laparoscopic sigmoid resection or conservative treatment. The randomization sequence with variable block size (2, 4, or 6) was generated using R statistical software version 3.0.0 with the Blockrand 1.1 package (R Foundation for Statistical Computing). The randomization sequence was stratified according to the inclusion criteria (recurrent, complicated, or persistent pain). The recruiters, health care professionals, outcome assessors, data collectors, and patients were unaware of the randomization sequence. Randomization and allocation to a treatment group was carried out using a web-based service created by the authors. All eligible patients were entered into a web-based electronic case report form. Due to the nature of the interventions, the LASER trial was an open-label trial. The patients, health care professionals, outcome assessors, and data analyzers were not blinded to the allocated intervention.

### Procedures

Patients randomized to conservative treatment received standardized written information regarding diverticulosis and constipation, were advised to increase the fiber content in their diet, and were prescribed a fiber supplement (eAppendixes 1-4 in Supplement 2). Patients allocated to elective laparoscopic sigmoid resection were scheduled for surgery within 3 months of randomization. After surgery, the patients were given the same standardized written information as the patients in the conservative treatment arm. The elective laparoscopic sigmoid resection was standardized as follows. Location of trocars was left to the decision of the operating surgeon. The distal transection line was in the upper rectum, below the promontory. The proximal transection line was in the proximal sigmoid colon or in the descending colon. Splenic flexure was mobilized unless a tension-free anastomosis could be created without mobilization. Inferior mesenteric and superior rectal arteries were left intact unless a tension-free anastomosis required division of the vessels. A circular stapler was used for colorectal anastomosis. Conservative treatment in the conservative treatment group was scheduled to continue for at least 6 months from randomization, unless an absolute indication for surgery emerged (such as fistula, stricture, or perforation). Patients were allowed to withdraw their consent to participate in the trial at any time, after which their data could no longer be collected, and they were treated according to normal clinical practice.

### Outcomes

The primary outcome of the trial was the difference in Gastrointestinal Quality of Life Index (GIQLI) score at randomization and at 6 months. GIQLI consists of 36 questions on gastrointestinal symptoms, each of which is scored from 0 to 4. The total range for GIQLI score is 0 to 144 points. Secondary outcomes were GIQLI score at 12, 24, 48, and 96 months; 36-Item Short Form Health Survey (SF-36) scores at 6, 12, 24, 48, and 96 months; recurrence and severity of recurrent diverticulitis (Hinchey classification); emergency surgery due to diverticulitis; elective sigmoid resection in the patients allocated to conservative treatment; complications due to elective sigmoid resection; mortality for any reason; complications of diverticular disease; and stoma rate. For both GIQLI and SF-36, a higher score indicated better QOL. Secondary outcomes up to the 6-month follow-up are reported here. Patients were contacted by phone and mail at 6 months and thereafter were contacted or will be contacted by mail at 12, 24, 48, and 96 months. If the patients did not respond to the letter or if the answers in the questionnaire were unclear, the patients were contacted by phone. The data were collected prospectively using electronic case report forms.

### Statistical Analysis

Based on earlier studies, in which mean preoperative GIQLI scores ranged from 95 to 100 and mean postoperative GIQLI scores ranged from 112 to 114,^15,16^ we aimed to show a difference in change of at least 12 GIQLI points between the groups. The minimal clinically important difference for GIQLI scores ranges from 6.42 to 7.64 points depending on the report.^20^ SD was assumed to be 22 points in both groups. Sample size calculations were done using 2-sided *t* tests for 2 independent means. A total of 120 patients were needed to show this difference with 90% power at 5% significance level (G*Power version 3.1.5.1). We assumed that up to 10% of patients could be lost to follow-up, yielding a final sample size of 133. A prespecified interim analysis was planned when the primary outcome could be assessed in 66 patients. The protocol stated that if a statistically significant difference could be detected in the primary outcome measure, the trial would be prematurely stopped (Supplement 1). The primary outcome and continuous secondary outcomes that were normally distributed (GIQLI score at 6 months) were compared using *t* test and effect size was reported as mean difference with 95% CI. Continuous secondary outcomes that were not normally distributed (physical component score and mental component score at 6 months)^21^ were compared using the Mann-Whitney *U* test and effect size was calculated as *r* = *Z*/√*N* without 95% CIs. If the GIQLI questionnaire was at least 75% complete, multiple imputation of the missing items was performed. Isolated items were missing from 3 patients at baseline and 8 patients at 6 months. Except for GIQLI score analysis, patients with missing data were omitted from analyses. Categorical secondary outcomes were compared using Fischer exact test (if expected cases in one cell were less than 5) or χ^2^ test and effect sizes were reported as odds ratios (ORs) with 95% CIs. Significance was set at a *P* value less than .05, and all *P* values were 2-tailed. Analyses were performed using SPSS version 25 (IBM). All outcomes were analyzed using the intention-to-treat principle where patients were analyzed within the group to which they had been randomized.

## Results

Between September 29, 2014, and October 10, 2018, 128 patients were assessed for eligibility, of whom 90 were enrolled and randomly assigned either to surgery (n = 45) or conservative treatment (n = 45) (Figure). The prespecified interim analysis was undertaken when 66 patients had been randomized and their 6-month follow-up data was assessable. Because of a significant difference in the primary outcome in this interim analysis, the trial recruiting was prematurely stopped. Because of the 6-month follow-up time required for the assessment of the results of these 66 patients, an additional 24 patients had already been recruited during this lag period, and they were included in the final analyses.

**Figure.**
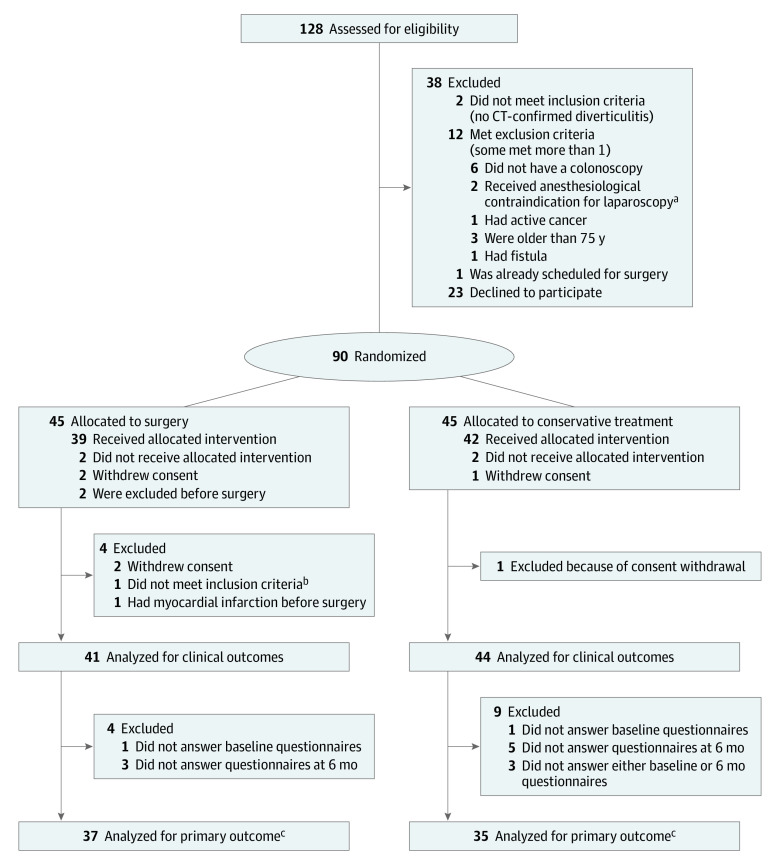
CONSORT Flow Chart CT indicates computed tomography. ^a^One because of severe obesity and one because of myasthenia gravis. ^b^Did not meet inclusion criteria (right-sided diverticulitis). ^c^Completed both baseline and 6-month quality of life questionnaires.

After exclusions, a total of 85 patients (26 male [31%]; mean [SD] age, 53.62 years [11.90]; 59 female [69%]; mean [SD] age, 57.08 [7.68]) were included in the intention-to-treat analysis (41 in the surgery group and 44 in the conservative treatment group) (Figure). Of these, 2 patients in the surgery group and 1 in the conservative treatment group withdrew their consent; 1 patient in the surgery group did not meet inclusion criteria and was excluded; and 1 patient in the surgery group was unable to undergo surgery because of a myocardial infarction and was excluded. In addition, 2 patients randomized to conservative treatment underwent elective sigmoid resection within 6 months, 1 because of recurrent diverticulitis and 1 because of chronic pain. Both were included in analyses in the conservative treatment group according to the intention-to-treat principle. Baseline characteristics are shown in Table 1. Mean (SD) body mass index was 29.3 (4.7) in the surgical group and 28.7 (4.2) in the conservative treatment group.

**Table 1.** Baseline Characteristics

Characteristic	No. (%)	
	Surgery (n = 41)	Conservative treatment (n = 44)
Age, median (IQR), y	59.0 (51.5-63.0)	59.0 (50.3-62.8)
Sex, male	11 (27)	15 (34)
Body mass index, mean (SD)^a^	29.3 (4.7)	28.7 (4.2)
Comorbidities		
Coronary disease/myocardial infarction	0	1 (2)
Congestive heart failure	0	1 (2)
Atrial fibrillation	1 (2)	1 (2)
Hypertension	15 (41)	11 (25)
Peripheral vascular disease	0	1 (2)
Cerebrovascular disease	0	0
Hemiplegia	0	0
Dementia	0	0
COPD or asthma	3 (7)	4 (9)
Connective tissue disease	3 (7)	3 (7)
Liver disease	0	0
Peptic ulcer	0	0
Diabetes	1 (2)	5 (11)
Without complications	1 (2)	4 (9)
With complications	0	1 (2)
Kidney disease (moderate/severe)	0	0
Cancer	0	0
Leukemia	0	0
Lymphoma	0	0
AIDS	0	0
Inclusion criteria^b^		
Recurrent diverticulitis	34 (83)	32 (73)
Complicated diverticulitis^c^	10 (24)	13 (30)
Persistent pain >3 mo after diverticulitis	2 (5)	3 (7)
Frequency of pain at randomization		
Never	4 (10)	3 (7)
Once a month	12 (29)	7 (16)
Once a week	7 (17)	8 (18)
A few times a week	8 (20)	9 (20)
Every day	1 (2)	5 (11)
Several times a day	0	1 (2)
All the time	2 (5)	1 (2)
VAS score, mean (SD)	4.2 (2.9)	4.6 (2.6)
GIQLI score at randomization, mean (SD)	104.75 (21.87)	99.75 (20.85)
SF-36 score at randomization, median (IQR)		
PCS	48.13 (42.42-53.50)	43.38 (36.73-49.29)
MCS	55.09 (44.89-60.15)	49.17 (40.70-57.64)
Episodes of diverticulitis, mean (SD)	4.6 (3.5)	4.0 (3.1)
Most severe diverticulitis before randomization		
Hinchey grade 0	7 (17)	11 (25)
Hinchey grade Ia	23 (56)	19 (43)
Hinchey grade Ib	5 (12)	8 (18)
Hinchey grade II	3 (7)	5 (11)
Hinchey grade III	2 (5)	0
Most invasive treatment for earlier diverticulitis		
Symptomatic treatment	1 (2)	1 (2)
Antibiotics	30 (73)	29 (66)
Percutaneous drainage	6 (15)	9 (20)
Laparoscopic lavage	2 (5)	1 (2)
Location of diverticulosis		
Whole colon	3 (7)	5 (11)
Sigmoid	24 (59)	24 (55)
Sigmoid and transverse	5 (12)	1 (2)
Descending colon	9 (22)	14 (32)
Earlier treatment of diverticulosis^d^		
No treatment	11 (27)	10 (23)
Multiple courses of per-oral antibiotics	23 (56)	25 (57)
Fiber supplement	22 (54)	24 (55)
Behavioral^e^	18 (44)	14 (32)
Medication		
Anticoagulative medication	3 (7)	1 (2)
Corticosteroid medication	1 (2)	1 (2)
Immunosuppressive medication	3 (7)	3 (7)

Abbreviations: COPD, chronic obstructive pulmonary disease; GIQLI, Gastrointestinal Quality of Life Index; IQR, interquartile range; MCS, mental component score; PCS, physical component score; SF-36, 36-Item Short Form Health Survey; VAS, visual analog scale.

^a^ Calculated as weight in kilograms divided by height in meters squared.

^b^ Patients may have met more than 1 inclusion criteria.

^c^ Complicated diverticulitis was defined as patients having 1 or more abscesses (10 in surgery group and 13 in conservative treatment group).

^d^ More than 1 form of treatment could be assigned per patient.

^e^ Behavior treatments included diet changes (higher levels of fiber intake) for treatment of constipation if necessary.

All elective sigmoid resections were started laparoscopically. Because of abundant intra-abdominal adhesions, 1 operation in the surgery group had to be converted to open surgery (Table 2). Moreover, 2 patients randomized to surgery had postoperative abscesses that were percutaneously drained, and 2 patients randomized to surgery had anastomotic leakage leading to emergency laparotomy and transversostomy in one patient and emergency laparoscopic lavage and drainage with transversostomy in the other. In these patients, the stomas were reversed after 7 and 2.5 months, respectively. This resulted in a 10% rate of Clavien-Dindo grade III complications in the surgery group. Otherwise, postoperative complications were minor. There was no mortality within 90 days of surgery (Table 2). None of the patients in the conservative treatment group had stoma within 6 months.

**Table 2.** Operative Characteristics

Characteristic	No. (%)	
	Surgery (n = 41)	Conservative treatment (n = 44)
Surgery		
Laparoscopy	38 (93)	2 (5)
Conversion to open surgery	1 (2)	0
Open	0	0
Stoma in primary operation	0	0
Complications (Clavien-Dindo grade)		
I	9 (22)	0
Dermatitis	1 (2)	0
Pain	2 (5)	0
Seroma	1 (2)	0
Hematuria	1 (2)	0
Fever	1 (2)	0
Thrombophlebitis	1 (2)	0
Nausea	1 (2)	0
Superficial wound infection	1 (2)	0
II	2 (5)	0
Urinary tract infection	1 (2)	0
Anastomotic intraluminal bleeding	1 (2)	0
IIIa	2 (5)	0
Abscess (percutaneous drainage)	2 (5)	0
IIIb	2 (5)	0
Anastomotic leakage requiring reoperation	2 (5)	0
IV	0	0
90-d Mortality	0	0
Stoma due to complications		
Temporary	2 (5)	0
Permanent	0	0

A total of 72 patients (37 in the surgery group and 35 in the conservative treatment group) answered both the baseline and 6-month QOL questionnaires and were included in analyses for the primary outcome (Figure). The primary outcome, the difference in GIQLI score between baseline and 6 months from randomization, was a mean of 11.96 points higher in the surgical group compared with the conservative treatment group (mean [SD] of 11.96 [15.89] points vs −0.2 [19.07] points; difference, 11.96; 95% CI, 3.72-20.19; *P* = .005) (Table 3). Similarly, the crude GIQLI score at 6 months was higher in the surgical group compared with the conservative treatment group (mean [SD] of 114.92 [16.77] points vs 101.97 [21.74] points; difference, 12.95; 95% CI, 3.98-21.92; *P* = .005). The mental component score of SF-36 was higher in the surgical group compared with the conservative treatment group (median [interquartile range] of 55.14 [51.38-57.24] points vs 47.68 [41.27-56.82]; *P* = .02), but the physical component score of SF-36 was similar between the groups (median [interquartile range] of 51.52 [41.74-55.74] points vs 42.19 [35.67-53.76]; *P* = .06) (Table 3).

**Table 3.** Primary and Secondary End Points Within 6 Months

End point	Surgery (n = 41)	Conservative treatment (n = 44)	Effect size (95% CI)	*P* value
**Primary outcome**				
MD in GIQLI score between baseline and 6 mo, mean (SD)^a^	11.76 (15.89)	−0.2 (19.07)	MD = 11.96 (3.72-20.19)	.005
**Secondary outcomes**				
GIQLI score at 6 mo^b^	114.92 (16.77)	101.97 (21.74)	MD = 12.95 (3.98-21.92)	.005
SF-36 at 6 mo, median (IQR)^c^				
PCS	51.52 (41.74-55.74)	42.19 (35.67-53.76)	*r* = 0.215^d^	.06
MCS	55.14 (51.38-57.24)	47.68 (41.27-56.82)	*r* = 0.267^d^	.02
Patients with recurrent episodes of diverticulitis within 6 mo^e^				
Any	2 (5%)	12 (27%)	OR = 8.0 (1.7-38.8)	.004
Hinchey I	2 (5%)	12 (27%)	NA	
Hinchey IIa	0	0	NA	
Hinchey IIb	0	0	NA	
Hinchey III	0	0	NA	
Hinchey IV	0	0	NA	
Stoma				
Permanent	0	0	NA^f^	>.99
Temporary	2 (5%)	0	NA^f^	.23
Mortality within 6 mo	0	0	NA^f^	>.99

Abbreviations: GIQLI, Gastrointestinal Quality of Life Index; MCS, mental component score; MD, mean difference; NA, not applicable; OR, odds ratio; PCS, physical component score; SF-36, 36-Item Short Form Health Survey.

^a^ Data missing in 4 patients in the surgery group and 9 patients in the conservative treatment group.

^b^ Data missing in 3 patients in the surgery group and 8 patients in the conservative treatment group.

^c^ Data missing in 4 patients in the surgery group and 6 patients in the conservative treatment group.

^d^ Effect size is given here as *r* = *Z*/√*N* without 95% CI.

^e^ Data missing in 3 patients in the surgery group and 5 patients in the conservative treatment group.

^f^ Effect size could not be calculated because of a value of zero in one cell.

Within 6 months of randomization, 12 patients in the conservative treatment group had recurrent diverticulitis compared with 2 patients in the surgical group (Table 3). All recurrent episodes were Hinchey grade Ia. No complications of diverticulitis, such as abscess, fistula, stenosis, or bleeding, were noticed in either group. None of the patients in either group needed emergency surgery for diverticulitis. Among patients with diverticulosis extending beyond sigmoid and descending colon, 0 of 8 patients in the surgical group and 1 of 6 patients (17%) in the conservative treatment group had recurrence within 6 months. Among patients with diverticulosis confined to sigmoid and/or descending colon, 2 of 30 patients (7%) in the surgical group and 12 of 33 patients (36%) in the conservative treatment group had recurrence within 6 months.

Patients in both groups were similarly satisfied with their treatment. However, at 6 months, the patients in the conservative treatment group reported experiencing pain more often than those in the surgery group (Table 4).

**Table 4.** Patient Perception and Pain at 6 Months From Randomization

Outcome	No. (%)		*P* value
	Surgery (n = 41)	Conservative treatment (n = 44)	
Patient satisfaction with assigned treatment^a^			
Satisfied	32 (78)	30 (68)	.23
Not satisfied	1 (2)	5 (11)	
Could not tell	1 (2)	2 (5)	
Pain at 6 mo^b^	19 (46)	30 (68)	
Once a month	7 (17)	13 (30)	.04
Once a week	8 (20)	11 (25)	
A few times a week	2 (5)	3 (7)	
Every day	0	2 (5)	
Several times a day	1 (2)	1 (2)	
All the time	1 (2)	0	
VAS score at 6 mo, mean (SD)^c^	1.8 (1.9)	3.3 (2.0)	.002

Abbreviation: VAS, visual analog scale.

^a^ Seven patients in the surgery group and 7 in the conservative treatment group did not respond.

^b^ Five patients in the surgery group and 10 in the conservative treatment group did not respond.

^c^ Five patients in the surgery group and 9 in the conservative treatment group did not respond.

## Discussion

In the LASER trial, we randomly assigned patients with recurrent, complicated, or persistent painful diverticulitis to receive either elective laparoscopic sigmoid resection or conservative treatment. QOL of patients in the surgical group increased significantly more within 6 months and they reported fewer episodes of recurrent diverticulitis and less pain in terms of both frequency and severity at 6 months compared with patients in the conservative treatment group. Only 2 patients in the conservative treatment group underwent elective surgery within 6 months and none required emergency surgery.

Several retrospective studies have focused on evaluating the need for elective sigmoid resection based on the risk of future complications and emergency surgery.^22^ Some have tried to assess the benefit of elective sigmoid resection for patients with recurrent or complicated diverticulitis in terms of patient-related outcomes. These retrospective series have suggested that elective sigmoid resection improves QOL and reduces pain and the risk of recurrent diverticulitis.^16,23,24^ However, there have been no head-to-head comparisons of surgery vs conservative treatment^24^ until very recently,^18^ and the role of elective sigmoid resection in the improvement of symptoms is debatable, as symptoms might decrease and QOL increase even without treatment. In addition to the current study, to our knowledge, only 1 randomized clinical trial comparing elective sigmoid resection with conservative treatment has been published (DIRECT trial).^18^ Our trial is similar to the DIRECT trial, the main difference being that the LASER trial included patients whose diverticular abscess had been conservatively treated whereas the DIRECT trial did not include such patients. The results of the DIRECT and LASER trials were also quite similar. QOL in the surgical groups, as measured using GIQLI score, was 114.9 in the LASER trial and 114.4 in the DIRECT trial, and in the conservative treatment groups was 101.97 in the LASER trial and 100.4 in the DIRECT trial. Results regarding SF-36 QOL were mixed, as we reported improvement in the mental component score part of the questionnaire in the surgical group, while in the DIRECT trial, the physical component score improved in the surgical group.

There are some important differences between the trials. First, most of the patients in the LASER trial (66 [78%]) had recurrent diverticulitis and only a minority (5 [6%]) had persistent pain. In the DIRECT trial, most patients (69 [63%]) had ongoing complaints and a minority (40 [37%]) had recurrent diverticulitis. Second, only 2 patients (4%) randomized to conservative treatment crossed over to surgery within 6 months in the LASER trial compared with 13 of 56 patients (23%) in the DIRECT trial. Third, the stoma rate in the LASER trial was low (2 [5%]), and the stomas were reversed within 7 months. In the DIRECT trial’s surgical group, 10 patients (21%) required a stoma, and most were reversed at 6 months. Fourth, complications in the surgery group in the LASER trial were rare, and only 4 patients (10%) needed reintervention compared with 13 patients (28%) in the DIRECT trial. These differences might be explained by differences in patient characteristics. However, the cohorts of the LASER and DIRECT trials seem highly similar in terms of median age (LASER, 59 years in both groups; DIRECT, 54 years in the surgical group and 56 years in the conservative treatment group), sex (LASER, 11 of 41 were male [27%] in the surgical group and 15 of 44 were male [34%] in the conservative treatment group; DIRECT, 15 of 53 were male [28%] in the surgical group and 24 of 56 were male [43%] in the conservative treatment group), and mean (SD) body mass index (calculated as weight in kilograms divided by height in meters squared; LASER, 29.3 [4.7] in the surgical group and 28.7 [4.2] in the conservative treatment group; DIRECT, 28.7 [4.7] in the surgical group and 27.8 [4.9] in the conservative treatment group). It is interesting to note that although QOL differed significantly between surgery and conservative treatment groups, the patients reporting being similarly satisfied with the allocated treatment. Finally, costs are likely to be higher in the surgical group initially, but whether the costs even out in long-term follow-up remains to be explored.

### Limitations

Our trial has limitations. First, the trial was prematurely terminated, and the number of patients was relatively low. However, the termination was in agreement with the criteria prespecified in the study protocol. As it was already evident midtrial that elective sigmoid resection carries a clear QOL benefit, it would have been unethical to continue randomizing patients. The premature stopping of the trial might have affected the power of the outcomes. Also, the recruiting process was relatively slow and took more than 4 years to finish in 6 hospitals. Second, the trial did not include sham surgery to mask the intervention because we considered that this would make recruitment even more difficult. Thus, we cannot rule out a possible placebo effect in QOL results. One-third of the patients in the conservative treatment group reported recurrent diverticulitis within 6 months, which is a slightly higher rate compared with patients who have had 3 or more episodes of diverticulitis during their lifetime.^12^ Third, the inclusion criteria were relatively strict as patients had to have had at least 3 episodes of diverticulitis within 2 years to meet the criteria for recurrent diverticulitis. It is unclear whether surgery would be beneficial for patients who have less frequent episodes of diverticulitis. Fourth, the patients included had various indications for surgery (recurrence, abscess, or persistent pain), but most had recurrent diverticulitis. The number of patients recruited due to 1 episode of complicated diverticulitis or persistent pain after 1 episode of diverticulitis was small and prevented us from doing a subgroup analysis. Fifth, as with many questionnaire-based studies, some patients did not respond to QOL questionnaires, and thus the primary outcome was not assessable in all randomized patients, which could have introduced bias in the results.

## Conclusions 

In conclusion, our results indicated that laparoscopic sigmoidectomy for patients with either recurrent, complicated diverticulitis or persistent pain after diverticulitis was effective and improved QOL within 6 months but carried a 10% risk of major complications. Further outcomes of the LASER trial will be reported when longer follow-up data are assessable.

## Notes

Supplement 1. Trial protocol.

Supplement 2. eAppendix 1. Suolen umpipussitauti (divertikuloosi ja divertikuliitti). eAppendix 2. Diverticular disease (diverticulosis and diverticulitis). eAppendix 3. Ummetus. eAppendix 4. Constipation.

Supplement 3. Data sharing statement.
