# Supplementary material for: Comparing Laparoscopic Elective Sigmoid Resection With Conservative Treatment in Improving Quality of Life of Patients With Diverticulitis: The Laparoscopic Elective Sigmoid Resection Following Diverticulitis (LASER) Randomized Clinical Trial
Source: JAMA Surg. 2020 Nov 18;156(2):1–9. doi: 10.1001/jamasurg.2020.5151 (PMC7675217; doi:10.1001/jamasurg.2020.5151)
Supplement: Supplement 1. — Trial protocol. [file jamasurg-e205151-s001.pdf]

**LASER trial. LAparoscopic elective Sigmoid resection following divERticulitis - A multicenter, prospective, randomized clinical trial.**

Finnish name: Laparoskooppinen elektiivinen sigmoidresektio divertikuloosin vuoksi - randomoitu prospektiivinen monikeskustutimus

***(English translation of original study protocol in Finnish)***

Research plan v.1.6 (19.3.2014)

Alex Santos[1], Panu Mentula[1], Suvi Rasilainen[2], Tero Rautio[3], Arto Turunen[4], Samuli Aho[5], Tarja Pinta[6], Mikael Victorzon[7], Jukka Karvonen[8], Mirjami Uotila-Nieminen[9], Eero Kangas[10], Juha Rinne[11], Anna Fagerström[12], Tom Scheinin[1], Ville Sallinen[1]; LASER trial study group

LASER trial study group members: Matti Kairaluoma[5], Anu Erlich[5], Jan Andersén[7], Hillar Jääger[7], Heini Savolainen[12], Selja Koskensalo[1], Olli Kruuna[1], Heikki Huhtinen[8], Pirita Varpe[8]

[1] Helsinki University Central Hospital, Surgical Hospital

[2] Helsinki University Central Hospital, Jorvi Hospital

[3] Oulu University Hospital

[4] Kanta-Häme Central Hospital

[5] Central Finland Central Hospital

[6] South Ostrobothnia Central Hospital

[7] Vaasa Central Hospital

[8] Turku University Central Hospital

[9] North Karelia Central Hospital

[10] South Karelia Central Hospital

[11] Päijät-Häme Central Hospital

[12] Kuopio University Hospital

## Background

Diverticulosis is an increasingly common ailment in the aging population. An estimated 25% develop acute diverticulitis (Biondo et al., 2012). The prognosis of acute diverticulitis is related to its severity measured according to the Hinchey classification. The mildest diverticulitis heals on its own, while mortality from diverticulitis that causes fecal peritonitis is high (Wasvary et al., 1999). Peritoneal inflammation caused diverticulitis usually leads to an emergency sigmoid resection and possible intestinal contents diversion through the stoma. In contrast, milder forms of acute diverticulitis are usually treated conservatively with an intravenous antibiotic and abscesses are drained.

It has previously been recommended that patients with recurrent diverticulitis undergo elective sigmoid resection to avoid further problems with complicated diverticulitis (Rafferty et al., 2006). However, retrospective work has found that the most difficult diverticulitis episode is usually the very first one, and the occurrence of complicated diverticulitis decreases with the recurrence of diverticulitis (Chapman et al., 2006; Ritz et al., 2011). Second, elective surgery for complicated diverticulitis (e.g., abscess) has been recommended (Rafferty et al., 2006). However, this recommendation has also recently been questioned (Gaertner et al., 2013). On the other hand, elective sigmoid resection has been shown to improve quality of life in selected patients with either recurrent diverticulitis or complicated diverticulitis (Forgione et al., 2009; Pasternak et al., 2011).

Current knowledge of who should have elective sigmoid resection is very limited, is based on retrospective comparative series or prospective studies that lack a control group. To date, no randomized, prospective study comparing surgical treatment to conservative treatment in patients with a relative indication (recurrent diverticulitis, complicated diverticulitis, pain after acute diverticulitis) to elective sigmoid resection has been published. An extensive recent treatment recommendation for diverticulitis cannot provide definitive recommendations for the selection of patients for elective sigmoid resection due to the lack of randomized work (Andersen et al., 2012).

The main objective of the study is to investigate the effect of elective sigmoid resection on gastrointestinal quality of life by comparing surgical treatment to conservative treatment in a randomized prospective multicenter study. The secondary objective is to investigate recurrence, mortality, and surgical complications of diverticulitis.

## Hypothesis

Elective sigmoid resection improves quality of life and reduces recurrence of diverticulitis in follow-up.

## Procedures

**The study sites**

Helsinki University Central Hospital, Surgical Hospital

Helsinki University Central Hospital, Jorvi Hospital

Oulu University Hospital

Kanta-Häme Central Hospital

Central Finland Central Hospital

South Ostrobothnia Central Hospital

Vaasa Central Hospital

Turku University Central Hospital

North Karelia Central Hospital

South Karelia Central Hospital

Päijät-Häme Central Hospital

Kuopio University Hospital

## Intervention Groups

1. Selective sigmoid resection

2. Conservative treatment (lifestyle guidelines, fiber supplement)

## Study Patients

Inclusion criteria

1. Repeat left-side colon diverticulitis (at least 3, within a 2 years period, at least one CT confirmed)

2. Previously complicated diverticulitis * (excluding diverticulitis with only pericolic air)

3. Pain or persistent change in bowel movements after CT-confirmed left side diverticulitis of colon, which lasted for more than 3 months after acute treatment

* Complicated diverticulitis refers to acute left side diverticulitis verified by computed tomography that is associated with fistula, stricture, intestinal abscess or free air outside the intestinal lumen.

Exclusion criteria

1. Multimorbidity that prevents consideration for elective surgery

2. Contraindication to laparoscopy (e.g. anesthetic reason)

3. Colon stricture

4. Fistula (colocutan, colovesical, colovaginal, etc.)

5. Active cancer

6. Previous resection of the left colon or rectum

7. Acute diverticulitis that has not resolved (inflammatory values need to have returned to normal and fever has stopped)

8. Colonoscopy / sigmoidoscopy or colon computed tomography (virtual colonoscopy) has not been performed within 2 years of randomisation

9. Age <18 or> 75 years

10. Pregnancy

11. Inability to answer quality of life surveys (eg dementia, psychiatric illness, etc.)

## Randomization

The patient is randomized 1: 1 for either elective sigmoid resection or conservative treatment. Patients are stratified based on inclusion criteria so that there are equal numbers of patients from each inclusion group in both randomization groups. Randomization is done using a computer centrally from the Helsinki University Central Hospital (HUCH). In practice, the person performing the randomisation connects to the HUCH server via the Internet, which provides the researcher with information about the patient's randomisation group. Randomization is performed at an outpatient visit, after the patient has first given written consent to participate in the study. Based on the group, treatment is selected, i.e. the patient is either placed in a surgical queue for elective sigmoid resection or given lifestyle instructions as well as instructions on how to use fiber supplementation. Upon randomization, the patient completes SF-36 and GIQLI quality of life questionnaires. Patients are analyzed according to the intention-to-treat principle according to the original randomization group.

## Intervention

1. Elective laparoscopic sigmoid resection

The patient is scheduled for elective laparoscopic sigmoid resection. Surgery is performed within a maximum of 3 months of randomisation. CRF # 3 is filled just before surgery (e.g., during a preoperative visit). CRF # 2 is filled as the patient is discharged after elective sigmoid resection. After the operation, the patient is given lifestyle instructions as well as a recommendation for the use of a fiber supplement.

Technique of laparoscopy

1. Port setting free
2. Distal resection site in the upper rectum, below the promontory

3. Proximal resection site in oral sigmoid or descending colon
4. Splenic flexure is mobilized as needed to obtain a tension-free anastomosis.
5. The aim is to keep the blood vessels as good as possible. A. mesenterica inferior and A. Rectalis superior are sought to be spared. However, if necessary, the blood vessels can be ligated, for example to provide a non-tight anastomosis or if the dissection conditions are difficult.
6. Bowel anastomosis with ILS.

2. Conservative treatment

Lifestyle guidelines in writing
- Duodecim Medical Publications Information to the patient: The intestinal diverticular disease, 12/11/2012 Pertti Black River (www.terveysportti.fi)
- Medical book Duodecim Information for the patient: Constipation 8.10.2012 Pertti Mustajoki (www.terveysportti.fi)
Recommendation on the use of fiber supplement.

Action in the event of elective sigmoid resection in a patient randomized to conservative treatment:

Conservative treatment should be tried for at least 6 months of randomisation (= until the first quality of life survey) before it can be concluded that it has failed and the patient referred to elective sigmoid resection. Of course, surgical treatment can be performed earlier if the patient has developed absolute indications for elective sigmoid resection (eg fistula, stricture). If a patient randomized to conservative treatment has to be treated with elective sigmoid resection, the patient should complete the GIQLI and SF-36 quality of life questionnaires at the time of surgery. After surgery, the patient is followed according to the original follow-up schedule (i.e., surveys just before surgery (preoperative visit, etc.), 6 months, 12 months, 24 months, 48 months, and 96 months after the initial randomisation). In accordance with normal research ethical principles, the patient may also opt out of the study altogether, at which point his or her follow-up will cease and he or she will be treated according to normal treatment lines and at the discretion of the attending physician.

## Outcomes

**Primary outcome**

Gastrointestinal Quality of Life Index (GIQLI) - difference between 0 months and 6 months from randomisation.

**Secondary variables**

1. Gastrointestinal Quality of Life Index (GIQLI) at 12 months, 24 months, 48 months and 96 months of randomisation.
2. SF-36 quality of life survey score at 6 months, 12 months, 24 months, 48 months and 96 months from randomisation.
3. Recurrence of diverticulitis and Hinchey class.
4. Undergoing emergency surgery due to diverticulosis / diverticulitis.
5. Undergoing elective sigmoid resection (conservative treatment group).
6. Complications of elective sigmoid resection.
7. Mortality (for any reason in both groups).
8. Complications of diverticulosis (fistula, stricture).
9. Stoma (permanent / temporary).

## Follow-up

The attached flow chart shows patient randomisation, timing of treatment procedures, and follow-up. Patients are called by phone 6 months after randomisation to inquire about well-being and a decision can be made on a possible change in treatment (a patient in the conservative treatment group can be referred for elective sigmoid resection).

Patients are also monitored by mailed questionnaires (SF-36, GIQLI, and a questionnaire prepared by researchers, see appendix) just before surgery (preoperative visit, etc.) and 6 months, 12 months, 24 months, 48 months, and 96 months after randomisation. The same questionnaires are also completed just before elective sigmoid resection. If there is any ambiguity in the answers to the questionnaire, the researcher will, if necessary, contact the patient by telephone to clarify the ambiguities.

The researcher at each center collects a questionnaire from his or her own center and also checks the entries in the patient records in his or her own area. Based on these, the researcher fills out a CRF # 3 form, which is therefore completed at each follow-up time point.

## Costs

The study is not expected to incur significant additional costs, as elective sigmoid resection is already a normal treatment and inclusion criteria follow general treatment recommendations. The only costs come

from the phone call made to the patient (at 6 months) and the postage costs of the questionnaires. Research may even save hospital costs, as half of patients are randomized to conservative care and no surgical costs are involved.

## Parameters to be collected

Case Report Form (see appendix). Case report form # 1 is completed at the time of randomization (outpatient visit). Case report from # 2 is completed electronically when the patient is discharged. Case report form # 3 is completed at each follow-up time point (just before surgery (preoperative visit, etc.), 6 months, 12 months, 24 months, 48 months, 96 months).

## The sample size

In previous publications, preoperative GIQLI has been 95-100 (SD 22) and postoperative GIQLI 112-114 (SD 19-22) (Forgione et al., 2009; Pasternak et al., 2011). The sample size calculation was made using these values, and the study aims to show a difference of 12 GIQLI points, the standard deviation being assumed to be 22 points in both groups. Using 0.05 alpha, 0.9 power, and 1: 1 allocation, a sample size per group of 60 patients is obtained. The sample size calculation was performed using G * Power 3.1.5.1 (comparison of means, two-tailed, A priori). Approximately 10% of patients are estimated to disappear at follow-up, bringing the final sample size to a total of 133 patients.

## Schedule, interim analysis and completion of the study

The study is planned to start in summer / autumn 2014. An interim analysis will be performed in the middle of the study, ie for 66 patients. If a statistically significant difference is obtained for the primary outcome already at this stage, the study will not be continued. Otherwise, the study will continue until 133 patients have been randomized. It is estimated that it will take about five years to recruit patients. The follow-up phase will continue for another eight years.

## Registration

The study is registered with clinicaltrials.gov before randomization begins.

## Privacy Policy

Personal data of randomized patients are collected in the study folder, and CRF # 1, # 2, and # 3 are completed for them. Patients who meet the inclusion criteria and thus are evaluated for participation in the study will be accounted for, how many patients will be excluded from the study, and for what reason

(fulfillment of the exclusion criterion, refusal, etc.). Personal data of patients who dropped out of the study will not be collected. The material to be collected is stored in a locked state and the electronic data is protected by passwords and encryption on computers.

## References


Andersen JC, Bundgaard L, Elbrønd H, Laurberg S, Walker LR, Støvring J, Danish Surgical Society. 2012. Danish national guidelines for treatment of diverticular disease. Dan Med J 59:C4453.

Biondo S, Lopez Borao J, Millan M, Kreisler E, Jaurrieta E. 2012. Current status of the treatment of acute colonic diverticulitis: a systematic review. Colorectal Disease 14:e1–e11.

Chapman JR, Dozois EJ, Wolff BG, Gullerud RE, Larson DR. 2006. Diverticulitis: a progressive disease? Do multiple recurrences predict less favorable outcomes? Ann Surg 243:876–830; discussion 880–3.

Forgione A, Leroy J, Cahill RA, Bailey C, Simone M, Mutter D, Marescaux J. 2009. Prospective Evaluation of Functional Outcome After Laparoscopic Sigmoid Colectomy. Ann Surg 249:218–224.

Gaertner WB, Willis DJ, Madoff RD, Rothenberger DA, Kwaan MR, Belzer GE, Melton GB. 2013. Percutaneous Drainage of Colonic Diverticular Abscess. Dis Colon Rectum 56:622–626.

Pasternak I, Wiedemann N, Basilicata G, Melcher GA. 2011. Gastrointestinal quality of life after laparoscopic-assisted sigmoidectomy for diverticular disease. Int J Colorectal Dis 27:781–787.

Rafferty J, Shellito P, Hyman NH, Buie WD. 2006. Practice Parameters for Sigmoid Diverticulitis. Dis Colon Rectum 49:939–944.

Ritz J-P, MD KSL, MD BF, PhD AS, FRCS HJBMF, MD CH. 2011. Outcome of patients with acute sigmoid diverticulitis: Multivariate analysis of risk factors for free perforation. Surgery 149:606–613.

Wasvary H, Turfah F, Kadro O, Beauregard W. 1999. Same hospitalization resection for acute diverticulitis. Am Surg 65:632–5– discussion 636.

**LASER trial. LAparoscopic elective Sigmoid resection following divERticulitis - A multicenter, prospective, randomized clinical trial.**

Finnish name: Laparoskooppinen elektiivinen sigmoidresektio divertikuloosin vuoksi - randomoitu prospektiivinen monikeskustutimus

***(English translation of original study protocol in Finnish)***

Research plan v.*1.7 (11.1.2017)* (changes in red)

Alex Santos[1], Panu Mentula[1], Suvi Rasilainen[2], Tero Rautio[3], Arto Turunen[4], Samuli Aho[5], Tarja Pinta[6], Mikael Victorzon[7], Jukka Karvonen[8], Mirjami Uotila-Nieminen[9], Eero Kangas[10], Juha Rinne[11], Anna Fagerström[12], Aleksi Lähdesmäki[13], Tom Scheinin[1], Ville Sallinen[1]; LASER trial study group

LASER trial study group members: Matti Kairaluoma[5], Anu Erlich[5], Risto Juusela[7], Jan Andersén[7], Hillar Jääger[7], Heini Savolainen[12], Selja Koskensalo[1], Olli Kruuna[1], Heikki Huhtinen[8], Pirita Varpe[8] Jussi Luhtala[3]

[1] Helsinki University Central Hospital, Surgical Hospital

[2] Helsinki University Central Hospital, Jorvi Hospital

[3] Oulu University Hospital

[4] Kanta-Häme Central Hospital

[5] Central Finland Central Hospital

[6] South Ostrobothnia Central Hospital

[7] Vaasa Central Hospital

[8] Turku University Central Hospital

[9] North Karelia Central Hospital

[10] South Karelia Central Hospital

[11] Päijät-Häme Central Hospital

[12] Kuopio University Hospital

[13] Hyvinkää Central Hospital

## Background

Diverticulosis is an increasingly common ailment in the aging population. An estimated 25% develop acute diverticulitis (Biondo et al., 2012). The prognosis of acute diverticulitis is related to its severity measured according to the Hinchey classification. The mildest diverticulitis heals on its own, while mortality from diverticulitis that causes fecal peritonitis is high (Wasvary et al., 1999). Peritoneal inflammation caused diverticulitis usually leads to an emergency sigmoid resection and possible intestinal contents diversion

through the stoma. In contrast, milder forms of acute diverticulitis are usually treated conservatively with an intravenous antibiotic and abscesses are drained.

It has previously been recommended that patients with recurrent diverticulitis undergo elective sigmoid resection to avoid further problems with complicated diverticulitis (Rafferty et al., 2006). However, retrospective work has found that the most difficult diverticulitis episode is usually the very first one, and the occurrence of complicated diverticulitis decreases with the recurrence of diverticulitis (Chapman et al., 2006; Ritz et al., 2011). Second, elective surgery for complicated diverticulitis (e.g., abscess) has been recommended (Rafferty et al., 2006). However, this recommendation has also recently been questioned (Gaertner et al., 2013). On the other hand, elective sigmoid resection has been shown to improve quality of life in selected patients with either recurrent diverticulitis or complicated diverticulitis (Forgione et al., 2009; Pasternak et al., 2011).

Current knowledge of who should have elective sigmoid resection is very limited, is based on retrospective comparative series or prospective studies that lack a control group. To date, no randomized, prospective study comparing surgical treatment to conservative treatment in patients with a relative indication (recurrent diverticulitis, complicated diverticulitis, pain after acute diverticulitis) to elective sigmoid resection has been published. An extensive recent treatment recommendation for diverticulitis cannot provide definitive recommendations for the selection of patients for elective sigmoid resection due to the lack of randomized work (Andersen et al., 2012).

The main objective of the study is to investigate the effect of elective sigmoid resection on gastrointestinal quality of life by comparing surgical treatment to conservative treatment in a randomized prospective multicenter study. The secondary objective is to investigate recurrence, mortality, and surgical complications of diverticulitis.

## Hypothesis

Elective sigmoid resection improves quality of life and reduces recurrence of diverticulitis in follow-up.

## Procedures

**The study sites**

Helsinki University Central Hospital, Surgical Hospital

Helsinki University Central Hospital, Jorvi Hospital

Oulu University Hospital

Kanta-Häme Central Hospital

Central Finland Central Hospital

South Ostrobothnia Central Hospital

Vaasa Central Hospital

Turku University Central Hospital

North Karelia Central Hospital

South Karelia Central Hospital

Päijät-Häme Central Hospital

Kuopio University Hospital

Hyvinkää Central Hospital

## Intervention Groups

1. Selective sigmoid resection

2. Conservative treatment (lifestyle guidelines, fiber supplement)

## Study Patients

Inclusion criteria

1. Repeat left-side colon diverticulitis (at least 3, within a 2 years period, at least one CT confirmed)

2. Previously complicated diverticulitis * (excluding diverticulitis with only pericolic air)

3. Pain or persistent change in bowel movements after CT-confirmed left side diverticulitis of colon, which lasted for more than 3 months after acute treatment

* Complicated diverticulitis refers to acute left side diverticulitis verified by computed tomography that is associated with fistula, stricture, intestinal abscess or free air outside the intestinal lumen.

Exclusion criteria

1. Multimorbidity that prevents consideration for elective surgery

2. Contraindication to laparoscopy (e.g. anesthetic reason)

3. Colon stricture

4. Fistula (colocutan, colovesical, colovaginal, etc.)

5. Active cancer

6. Previous resection of the left colon or rectum

7. Acute diverticulitis that has not resolved (inflammatory values need to have returned to normal and fever has stopped)

8. Colonoscopy / sigmoidoscopy or colon computed tomography (virtual colonoscopy) has not been performed within 2 years of randomisation

9. Age <18 or> 75 years

10. Pregnancy

11. Inability to answer quality of life surveys (eg dementia, psychiatric illness, etc.)

## Randomization

The patient is randomized 1: 1 for either elective sigmoid resection or conservative treatment. Patients are stratified based on inclusion criteria so that there are equal numbers of patients from each inclusion group in both randomization groups. Randomization is done using a computer centrally from the Helsinki University Central Hospital (HUCH). In practice, the person performing the randomisation connects to the HUCH server via the Internet, which provides the researcher with information about the patient's randomisation group. Randomization is performed at an outpatient visit, after the patient has first given written consent to participate in the study. Based on the group, treatment is selected, i.e. the patient is either placed in a surgical queue for elective sigmoid resection or given lifestyle instructions as well as instructions on how to use fiber supplementation. Upon randomization, the patient completes SF-36 and GIQLI quality of life questionnaires. Patients are analyzed according to the intention-to-treat principle according to the original randomization group.

## Intervention

1. Elective laparoscopic sigmoid resection

The patient is scheduled for elective laparoscopic sigmoid resection. Surgery is performed within a maximum of 3 months of randomisation. CRF # 3 is filled just before surgery (e.g., during a preoperative visit). CRF # 2 is filled as the patient is discharged after elective sigmoid resection. After the operation, the patient is given lifestyle instructions as well as a recommendation for the use of a fiber supplement.

Technique of laparoscopy

1. Port setting free
2. Distal resection site in the upper rectum, below the promontory
3. Proximal resection site in oral sigmoid or descending colon
4. Splenic flexure is mobilized as needed to obtain a tension-free anastomosis.
5. The aim is to keep the blood vessels as good as possible. A. mesenterica inferior and A. Rectalis superior are sought to be spared. However, if necessary, the blood vessels can be ligated, for example to provide a non-tight anastomosis or if the dissection conditions are difficult.
6. Bowel anastomosis with ILS.

2. Conservative treatment

Lifestyle guidelines in writing
- Duodecim Medical Publications Information to the patient: The intestinal diverticular disease, 12/11/2012 Pertti Black River (www.terveysportti.fi)
- Medical book Duodecim Information for the patient: Constipation 8.10.2012 Pertti Mustajoki (www.terveysportti.fi)

Recommendation on the use of fiber supplement.

Action in the event of elective sigmoid resection in a patient randomized to conservative treatment:

Conservative treatment should be tried for at least 6 months of randomisation (= until the first quality of life survey) before it can be concluded that it has failed and the patient referred to elective sigmoid resection. Of course, surgical treatment can be performed earlier if the patient has developed absolute indications for elective sigmoid resection (eg fistula, stricture). If a patient randomized to conservative treatment has to be treated with elective sigmoid resection, the patient should complete the GIQLI and SF-36 quality of life questionnaires at the time of surgery. After surgery, the patient is followed according to the original follow-up schedule (i.e., surveys just before surgery (preoperative visit, etc.), 6 months, 12 months, 24 months, 48 months, and 96 months after the initial randomisation). In accordance with normal research ethical principles, the patient may also opt out of the study altogether, at which point his or her follow-up will cease and he or she will be treated according to normal treatment lines and at the discretion of the attending physician.

## Outcomes

### Primary outcome

Gastrointestinal Quality of Life Index (GIQLI) - difference between 0 months and 6 months from randomisation.

### Secondary variables

1. Gastrointestinal Quality of Life Index (GIQLI) at 12 months, 24 months, 48 months and 96 months of randomisation.
2. SF-36 quality of life survey score at 6 months, 12 months, 24 months, 48 months and 96 months from randomisation.
3. Recurrence of diverticulitis and Hinchey class.
4. Undergoing emergency surgery due to diverticulosis / diverticulitis.
5. Undergoing elective sigmoid resection (conservative treatment group).
6. Complications of elective sigmoid resection.
7. Mortality (for any reason in both groups).
8. Complications of diverticulosis (fistula, stricture).
9. Stoma (permanent / temporary).

## Follow-up

The attached flow chart shows patient randomisation, timing of treatment procedures, and follow-up. Patients are called by phone 6 months after randomisation to inquire about well-being and a decision can be made on a possible change in treatment (a patient in the conservative treatment group can be referred for elective sigmoid resection).

Patients are also monitored by mailed questionnaires (SF-36, GIQLI, and a questionnaire prepared by researchers, see appendix) just before surgery (preoperative visit, etc.) and 6 months, 12 months, 24 months, 48 months, and 96 months after randomisation. The same questionnaires are also completed just before elective sigmoid resection. If there is any ambiguity in the answers to the questionnaire, the researcher will, if necessary, contact the patient by telephone to clarify the ambiguities.

The researcher at each center collects a questionnaire from his or her own center and also checks the entries in the patient records in his or her own area. Based on these, the researcher fills out a CRF # 3 form, which is therefore completed at each follow-up time point.

## Costs

The study is not expected to incur significant additional costs, as elective sigmoid resection is already a normal treatment and inclusion criteria follow general treatment recommendations. The only costs come from the phone call made to the patient (at 6 months) and the postage costs of the questionnaires. Research may even save hospital costs, as half of patients are randomized to conservative care and no surgical costs are involved.

## Parameters to be collected

Case Report Form (see appendix). Case report form # 1 is completed at the time of randomization (outpatient visit). Case report from # 2 is completed electronically when the patient is discharged. Case report form # 3 is completed at each follow-up time point (just before surgery (preoperative visit, etc.), 6 months, 12 months, 24 months, 48 months, 96 months).

## The sample size

In previous publications, preoperative GIQLI has been 95-100 (SD 22) and postoperative GIQLI 112-114 (SD 19-22) (Forgione et al., 2009; Pasternak et al., 2011). The sample size calculation was made using these values, and the study aims to show a difference of 12 GIQLI points, the standard deviation being assumed to be 22 points in both groups. Using 0.05 alpha, 0.9 power, and 1: 1 allocation, a sample size per group of 60 patients is obtained. The sample size calculation was performed using G * Power 3.1.5.1 (comparison of means, two-tailed, A priori). Approximately 10% of patients are estimated to disappear at follow-up, bringing the final sample size to a total of 133 patients.

## Schedule, interim analysis and completion of the study

The study is planned to start in summer / autumn 2014. An interim analysis will be performed in the middle of the study, ie for 66 patients. If a statistically significant difference is obtained for the primary outcome already at this stage, the study will not be continued. Otherwise, the study will continue until 133 patients have been randomized. It is estimated that it will take about five years to recruit patients. The follow-up phase will continue for another eight years.

## Registration

The study is registered with clinicaltrials.gov before randomization begins.

## Privacy Policy

Personal data of randomized patients are collected in the study folder, and CRF # 1, # 2, and # 3 are completed for them. Patients who meet the inclusion criteria and thus are evaluated for participation in the study will be accounted for, how many patients will be excluded from the study, and for what reason (fulfillment of the exclusion criterion, refusal, etc.). Personal data of patients who dropped out of the study will not be collected. The material to be collected is stored in a locked state and the electronic data is protected by passwords and encryption on computers.

## References


Andersen JC, Bundgaard L, Elbrønd H, Laurberg S, Walker LR, Støvring J, Danish Surgical Society. 2012. Danish national guidelines for treatment of diverticular disease. Dan Med J 59:C4453.

Biondo S, Lopez Borao J, Millan M, Kreisler E, Jaurrieta E. 2012. Current status of the treatment of acute colonic diverticulitis: a systematic review. Colorectal Disease 14:e1–e11.

Chapman JR, Dozois EJ, Wolff BG, Gullerud RE, Larson DR. 2006. Diverticulitis: a progressive disease? Do multiple recurrences predict less favorable outcomes? Ann Surg 243:876–830; discussion 880–3.

Forgione A, Leroy J, Cahill RA, Bailey C, Simone M, Mutter D, Marescaux J. 2009. Prospective Evaluation of Functional Outcome After Laparoscopic Sigmoid Colectomy. Ann Surg 249:218–224.

Gaertner WB, Willis DJ, Madoff RD, Rothenberger DA, Kwaan MR, Belzer GE, Melton GB. 2013. Percutaneous Drainage of Colonic Diverticular Abscess. Dis Colon Rectum 56:622–626.

Pasternak I, Wiedemann N, Basilicata G, Melcher GA. 2011. Gastrointestinal quality of life after laparoscopic-assisted sigmoidectomy for diverticular disease. Int J Colorectal Dis 27:781–787.

Rafferty J, Shellito P, Hyman NH, Buie WD. 2006. Practice Parameters for Sigmoid Diverticulitis. Dis Colon Rectum 49:939–944.

Ritz J-P, MD KSL, MD BF, PhD AS, FRCS HJBMF, MD CH. 2011. Outcome of patients with acute sigmoid diverticulitis: Multivariate analysis of risk factors for free perforation. Surgery 149:606–613.

Wasvary H, Turfah F, Kadro O, Beauregard W. 1999. Same hospitalization resection for acute diverticulitis. Am Surg 65:632–5– discussion 636.

**LASER trial. LAparoscopic elective Sigmoid resection following divERticulitis - A multicenter, prospective, randomized clinical trial.**

Suomenkielinen nimi: Laparoskooppinen elektiivinen sigmaresektio divertikuloosin vuoksi - randomoitu prospektiivinen monikeskustutimus

Tutkimussuunnitelma v.1.6 (19.3.2014)

Alex Santos[1], Panu Mentula[1], Suvi Rasilainen[2], Tero Rautio[3], Arto Turunen[4], Samuli Aho[5], Tarja Pinta[6], Mikael Victorzon[7], Jukka Karvonen[8], Mirjami Uotila-Nieminen[9], Eero Kangas[10], Juha Rinne[11], Anna Fagerström[12], Tom Scheinin[1], Ville Sallinen[1]; LASER trial study group

LASER trial study group members: Matti Kairaluoma[5], Anu Erlich[5], Jan Andersén[7], Hillar Jääger[7], Heini Savolainen[12], Selja Koskensalo[1], Olli Kruuna[1], Heikki Huhtinen[8], Pirita Varpe[8]

[1] Helsingin yliopistollinen keskussairaala, Kirurginen sairaala
[2] Helsingin yliopistollinen keskussairaala, Jorvin sairaala
[3] Oulun yliopistollinen sairaala
[4] Kanta-Hämeen keskussairaala
[5] Keski-Suomen keskussairaala
[6] Etelä-Pohjanmaan keskussairaala
[7] Vaasan keskussairaala
[8] Turun yliopistollinen keskussairaala
[9] Pohjois-Karjalan keskussairaala
[10] Etelä-Karjalan keskussairaala
[11] Päijät-Hämeen keskussairaala
[12] Kuopion yliopistollinen sairaala

## Tausta

Divertikuloosi on yhä yleisempi vaiva ikääntyvässä väestössä. Arviolta 25% sairastuu akuutiin divertikuliittiin (Biondo et al., 2012). Akuutin divertikuliitin prognoosi on sidoksissa sen vaikeusasteeseen Hinchey-luokituksen perusteella. Lievimmät divertikuliitit paranevat itsestään, kun taas mortaliteetti fekaalisen peritoniitin aiheuttaneeseen divertikuliittiin on korkea (Wasvary et al., 1999). Vatsakalvontulehduksen aiheuttaneet divertikuliitit leikataan yleensä päivystyksenä tehtävällä sigmaresektiolla ja mahdollisella suolen sisällön diversiolla avanteen kautta. Sen sijaan lievemmät akuutin divertikuliitin muodot hoidetaan yleensä konservatiivisesti suonensisäisellä antibiootilla ja absessit dreeneraten.

Aiemmin on suositeltu, että potilaille, joilla on toistuvia divertikuliitteja, tehtäisiin elektiivinen sigmaresektio, jotta voidaan jatkossa välttää komplisoituneen divertikuliitin ilmentyminen (Rafferty et al., 2006). Kuitenkin retrospektiivisissä töissä on todettu, että puhjennut divertikulitti on useimmiten ensimmäinen, ja komplisoituneen divertikuliitin ilmeneminen vähenee divertikuliittien uusiessa (Chapman et al., 2006; Ritz et al., 2011). Toisekseen on suositeltu komplisoituneen divertikuliitin (esim. paise) leikkaamista elektiivisesti (Rafferty et al., 2006). Myös tämä suositus on hiljattain kuitenkin

kyseenalaistettu (Gaertner et al., 2013). Toisaalta on osoitettu, että elektiivinen sigmaresektio parantaa elämänlaatua valikoiduilla potilailla, jotka ovat sairastaneet joko toistuvia divertikuliitteja tai komplisoituneen divertikuliitin (Forgione et al., 2009; Pasternak et al., 2011).

Tämänhetkinen tieto siitä kenelle elektiivinen sigmaresektio tulisi tehdä on erittäin rajoittunutta, ja perustuu retrospektiivisiin vertaileviin sarjoihin, tai prospektiivisiin tutkimuksiin, joista puuttuu verrokkiryhmä. Tällä hetkellä ei ole julkaistu yhtään randomoitua, prospektiivista tutkimusta, jossa verrattaisiin leikkaushoitoa konservatiiviseen hoitoon potilailla, joilla on relatiivinen indikaatio (toistuvat divertikuliitit, komplisoitunut divertikuliitti, kiputila akuutin divertikuliitin jälkeen) elektiiviseen sigmaresektioon. Laajassa tuoreessa divertikuliitin hoitosuosituksessa ei voida antaa definitiivisiä suosituksia potilaiden valikoimiseen elektiiviseen sigmaresektioon randomisoitujen töiden puutteen vuoksi (Andersen et al., 2012).

Tutkimuksen päätarkoituksena on selvittää elektiivisen sigmaresektion vaikutusta gastroenterologiseen elämänlaatuun (Gastrointestinal Quality of Life) vertailemalla leikkaushoitoa konservatiiviseen hoitoon randomoidussa prospektiivisessa monikeskustutkimuksessa. Toissijaisena tarkoituksena on selvittää divertikuliittien uusiutumista, kuolleisuutta sekä leikkauskomplikaatioita.

## Hypoteesi

Elektiivinen sigmaresektio parantaa elämänlaatua ja vähentää divertikuliittien uusiutumista seurannassa.

## Menetelmät

### Tutkimuspaikat

Helsingin yliopistollinen keskussairaala, Kirurginen sairaala
Helsingin yliopistollinen keskussairaala, Jorvin sairaala
Oulun yliopistollinen sairaala
Kanta-Hämeen keskussairaala
Keski-Suomen keskussairaala
Etelä-Pohjanmaan keskussairaala
Vaasan keskussairaala
Turun yliopistollinen keskussairaala
Pohjois-Karjalan keskussairaala
Etelä-Karjalan keskussairaala
Päijät-Hämeen keskussairaala
Kuopion yliopistollinen sairaala

### Interventioryhmät

1. Elektiivinen sigmaresektio
2. Konservatiivinen hoito (elämäntapaohjeet, kuitulisä)

### Tutkimuspotilaat

Inkluusiokriteerit

1. Toistuvat vas. puoleiset koolon divertikuliitit (vähintään 3, vähintään 2v sisällä, ainakin yksi TT:llä varmistettu)

2. Aiemmin sairastettu komplisoitunut divertikuliitti* (poislukien divertikuliitit, joissa vain perikoolista ilmaa)
3. TT:llä varmistetun vas. puoleisen koolon divertikuliitin jälkeiset kivut tai suolen toiminnan vaikeudet, jotka jatkuneet yli 3kk akuutin hoidon jälkeen

   * Komplisoituneella divertikuliitilla tarkoitetaan tietokonetomografialla verifioitua akuuttia vas. puoleista koolon divertikuliittia, johon liittyy fisteli, striktuura, absessi tai suolen ulkopuolella oleva tietokonetomografialla todettu ilma.

Ekskluusiokriteerit

1. Multimorbiditeetti, joka estää elektiivisen leikkausharkinnan
2. Este laparoskopialle (esim. anestesiologinen syy)
3. Koolonin striktuura
4. Fisteli (kolokutaani, kolovesikaali, kolovaginaali, tms)
5. Aktiivinen syöpä
6. Aiempi vasemman puoleisen koolonin tai rektumin resektio
7. Akuutti divertikuliitti, joka ei ole rauhoittunut (tulehdusarvot normaalistuneet, kuumeilu loppunut)
8. Kolonoskopiaa/sigmoideoskopiaa tai paksusuolen-tietokonetomografiaa (virtuaalikolonoskopiaa) ei ole tehty 2v sisällä randomisaatiosta
9. Ikä < 18 tai > 75 vuotta
10. Raskaus
11. Kyvyttömyys vastata elämänlaatukyselyihin (esim. dementia, psykiatrinen sairaus,tms)

**Satunnaistaminen**

Potilasta satunnaistetaan 1:1 joko elektiiviseen sigmaresektioon tai konservatiiviseen hoitoon. Potilaat satunnaistetaan stratifioidusti inkluusiokriteereiden perusteella, siten että molemmissa randomisaatioryhmissä olisi yhtä monta potilasta kustakin inkluusiokriteeriryhmästä. Satunnaistaminen tehdään tietokoneen avulla keskitetysti HYKS:stä käsin. Käytännössä randomisaation tekevä henkilö ottaa internetin välityksellä yhteyden HYKS:n palvelimeen, joka antaa tutkijalle tiedon potilaan randomisaatioryhmästä. Randomisaatio tehdään poliklinikkakäynnin yhteydessä, kun potilas on ensin antanut kirjallisen suostumuksen tutkimukseen osallistumiseen. Ryhmän perusteella valitaan hoito, eli potilas joko asetetaan leikkausjonoon elektiivista sigmaresektiota varten tai hänelle annetaan elämäntapaohjeistus sekä ohjeistus käyttää kuitulisää. Randomisaation yhteydessä potilas täyttää SF-36 ja GIQLI elämänlaatukyselykaavakkeet. Potilaat analysoidaan intention-to-treat periaatteen mukaisesti alkuperäisen randomisaatioryhmän mukaan.

**Interventio**

1. Elektiivinen laparoskooppinen sigmaresektio

   Potilas asetetaan leikkausjonoon elektiivistä laparoskooppista sigmaresektiota varten. Leikkaus suoritetaan **maksimissaan 3 kuukauden sisällä** randomisaatiosta. CRF #3 täytetään juuri ennen leikkausta (esim. preoperatiivisen

käynnin yhteydessä). CRF #2 täytetään potilaan kotiutuessa elektiivisen sigmaresektion jälkeen. Leikkauksen jälkeen potilaalle annetaan elämäntapaohjeistus sekä suositus kuitulisän käytöstä.

Tekniikka laparoskopia

1. Porttien asetus vapaa
2. Distaalinen katkaisukohta ylärektum, promontoriumin alapuolella
3. Proksimaalinen katkaisukohta oraalisessa sigmassa tai laskevassa koolonissa
4. Flexura lienalis mobilisoidaan tarvittaessa, jotta saadaan kiristyksetön sauma
5. Verisuonitus pyritään jättämään mahdollisimman hyväksi. A. mesenterica inferior ja a. rectalis superior pyritään säästämään. Tarvittaessa verisuonia voidaan kuitenkin katkaista esimerkiksi kiristyksettömän sauman aikaansaamiseksi tai mikäli dissektio-olosuhteeet ovat vaikeat.
6. Suolianastomoosi ILS-laitteella

2. Konservatiivinen hoito

Elämäntapaohjeistus kirjallisena
- Lääkärikirja Duodecim Tietoa potilaalle: Suolen umpipussi tauti, 12.11.2012 Pertti Mustajoki (www.terveysportti.fi)
- Lääkärikirja Duodecim Tietoa potilaalle: Ummetus 8.10.2012 Pertti Mustajoki (www.terveysportti.fi)

Suullinen suositus kuitulisän käytöstä.

Toiminta jouduttaessa elektiiviseen sigmaresektioon potilaalla, joka on randomoitu konservatiivisen hoidon ryhmään:

Konservatiivista hoitoa tulisi kokeilla vähintään 6kk randomisaatiosta (=ensimmäiseen elämänlaatukyselyyn saakka) ennen kuin voidaan päättää, että se on epäonnistunut ja potilas ohjata elektiiviseen sigmaresektioon. Luonnollisesti operatiivisen hoidon voi suorittaa aikaisemminkin, mikäli potilaalle on kehittynyt absoluuttisia indikaatioita elektiiviseen sigmaresektioon (esim. fisteli, striktuura). Mikäli potilas, joka on randomoitu konservatiiviseen hoitoon, joudutaan hoitamaan elektiivisella sigmaresektiolla, tulee potilaasta täyttää leikkauspäätöksen hetkellä GIQLI ja SF-36 elämänlaatukyselyt. Leikkauksen jälkeen potilasta seurataan alkuperäisen seurantascheeman mukaisesti (eli kyselyt juuri ennen leikkausta (preoperatiivinen käynti, tms.), 6kk, 12kk, 24kk, 48kk ja 96kk päästä **alkuperäisestä randomisaatiosta**). Normaalien tutkimuseettisten periaatteiden mukaisesti potilas voi myös itse jättäytyä kokonaan pois tutkimuksesta omasta halustaan, jolloin hänen seurantansa loppuu ja hänet hoidetaan normaalien hoitolinjojen ja hoitavan lääkärin harkinnan mukaisesti.

**Tutkittavat muuttujat**

Päävastemuuttuja

Gastrointestinal Quality of Life Index (GIQLI) - ero 0kk ja 6kk kohdalla randomisaatiosta

Toissijaiset muuttujat

1. Gastrointestinal Quality of Life Index (GIQLI) 12kk, 24kk, 48kk ja 96kk kohdalla randomisaatiosta
2. SF-36 elämänlaatukyselyn pistemäärä 6kk, 12kk, 24kk, 48kk ja 96kk kohdalla randomisaatiosta
3. Divertikuliittien uusiutuminen ja Hinchey-luokka
4. Joutuminen päivystykselliseen leikkaukseen divertikuloosin/divertikuliitin vuoksi
5. Joutuminen elektiiviseen sigmaresektioon (konservatiivinen hoitoryhmä)
6. Elektiivisesta sigmaresektiosta aiheutuneet komplikaatiot
7. Mortaliteetti (mistä tahansa syystä molemmissa ryhmissä)
8. Divertikuloosin komplikaatiot (fisteli, striktuura)
9. Avanne (pysyvä / väliaikainen)

**Seuranta**

Liitteenä olevasta vuokaaviosta käy ilmi potilaiden randomisaatio, hoitotoimenpiteiden ajoittaminen sekä seuranta.
Potilaille varataan soittoaika 6kk päähän randomisaatiosta, jossa tiedustellaan vointi, ja voidaan päättää mahdollisesta hoitotavan muutoksesta (konservatiivisen hoidon ryhmän potilas ohjata elektiiviseen sigmaresektioon).
Potilaita seurataan lisäksi postitse lähetettävin kyselykaavakkein (SF-36, GIQLI, sekä tutkijoiden laatima kyselykaavake, kts. liite) juuri ennen leikkausta (preoperatiivinen käynti, tms.) sekä 6kk, 12kk, 24kk, 48kk ja 96kk päästä randomisaatiosta. Samat kyselykaavakkeet täytetään myös juuri ennen elektiivistä sigmaresektiota. Mikäli kyselykaavakkeen vastauksissa on epäselvyyttä, tutkija ottaa tarvittaessa potilaaseen yhteyttä puhelimitse selvittääkseen epäselvyydet.
Kunkin keskuksen tutkija kerää oman keskuksensa kyselykaavakkeen ja tarkistaa tässä yhteydessä myös oman alueensa potilasasiakirjoista merkinnät. Näiden perusteella tutkija täyttää CRF #3-lomakkeen, joka täytetään siis jokaisessa seuranta-aikapisteessä.

**Kustannukset**

Tutkimuksen ei arvioida aiheuttavan merkittäviä ylimääräisiä kuluja, sillä elektiivinen sigmaresektio on normaalia hoitoa jo tällä hetkellä ja inkluusiokriteerit noudattavat yleisiä hoitosuosituksia. Ainoat kustannukset tulevat potilaalle tehtävästä puhelinsoitosta (6kk kohdalla) sekä kyselykaavakkeiden postituskustannuksista. Tutkimus saattaa jopa säästää sairaaloiden kuluja, sillä puolet potilaista randomoidaan konservatiiviseen hoitoon ja heidän osaltaan leikkauskustannukset puuttuvat.

**Kerättävät parametrit**

Case Report Form (kts. liite). Case report form #1 täytetään randomisaation yhteydessä (poliklinikkakäynti). Case report from #2 täytetään elektiivisesti leikatun potilaan

kotiutuessa. Case report form #3 täytetään jokaisessa seuranta-aikapisteessä (juuri ennen leikkausta (preoperatiivinen käynti, tms.), 6kk, 12kk, 24kk, 48kk, 96kk).

**Otoskoko**

Aikaisemmissa julkaisuissa preoperatiivinen GIQLI on ollut 95-100 (SD 22) ja postoperatiivinen GIQLI 112-114 (SD 19-22) (Forgione et al., 2009; Pasternak et al., 2011). Otoskokolaskelma tehtiin käytäen näitä arvoja, ja tutkimuksessa pyritään näyttämään 12 GIQLI-pisteen ero, standardideviaatioksi oleteteaan molemmissa ryhmissä 22 pistettä. Käyttäen 0.05 alfaa, 0.9 voimaa ja 1:1 allokaatiota saadaan otoskooksi per ryhmä 60 potilasta. Otoskokolaskema tehtiin käyttäen G*Power 3.1.5.1-ohjelmaa (comparison of means, two-tailed, A priori). Seurannassa arvioidaan häviävän noin 10% potilaista, joten lopulliseksi otoskooksi tulee yhteensä 133 potilasta.

**Aikataulu, välianalyysi ja tutkimuksen päättyminen**

Tutkimus suunnitellaan alkavan kesällä/syksyllä 2014. Tutkimuksen puolivälissä eli 66 potilaan kohdalla suoritetaan välianalyysi. Mikäli jo tässä vaiheessa saadaan päävastemuuttujaan tilastollisesti merkittävä ero, ei tutkimusta jatketa eteenpäin. Muuten tutkimus jatkuu, kunnes 133 potilasta on randomoitu. Potilaiden rekrytoimisen arvioidaan kestävän n. viisi vuotta. Seurantavaihe kestää tästä vielä kahdeksan vuotta eteenpäin.

**Rekisteröinti**

Tutkimus rekisteröidään ennen satunnaistamisten aloittamista clinicaltrials.gov-palveluun.

**Tietosuoja**

Randomoitujen potilaiden henkilötiedot kerätään tutkimuskansioon, sekä heistä täytetään CRF #1, #2 ja #3. Potilaista, jotka täyttävät inkluusiokriteerit ja siten arvioidaan tutkimukseen osallistumista, pidetään kirjanpitoa, siitä kuinka monta potilasta jää pois tutkimuksesta, ja mistä syystä (ekskluusiokriteerin täyttyminen, kieltäytyminen, tms). Tutkimuksesta poisjääneiden potilaiden henkilötietoja ei kerätä. Kerättävä aineisto säilytetään lukitussa tilassa ja sähköiset tiedot tietokoneilla salasanalla ja salauksella suojattuina.

**Viitteet**


Andersen JC, Bundgaard L, Elbrønd H, Laurberg S, Walker LR, Støvring J, Danish Surgical Society. 2012. Danish national guidelines for treatment of diverticular disease. Dan Med J 59:C4453.

Biondo S, Lopez Borao J, Millan M, Kreisler E, Jaurrieta E. 2012. Current status of the treatment of acute colonic diverticulitis: a systematic review. Colorectal Disease 14:e1–e11.

Chapman JR, Dozois EJ, Wolff BG, Gullerud RE, Larson DR. 2006. Diverticulitis: a progressive disease? Do multiple recurrences predict less favorable outcomes? Ann Surg 243:876–830; discussion 880–3.

Forgione A, Leroy J, Cahill RA, Bailey C, Simone M, Mutter D, Marescaux J. 2009. Prospective Evaluation of Functional Outcome After Laparoscopic Sigmoid Colectomy. Ann Surg 249:218–224.

Gaertner WB, Willis DJ, Madoff RD, Rothenberger DA, Kwaan MR, Belzer GE, Melton GB. 2013. Percutaneous Drainage of Colonic Diverticular Abscess. Dis Colon Rectum 56:622–626.

Pasternak I, Wiedemann N, Basilicata G, Melcher GA. 2011. Gastrointestinal quality of life after laparoscopic-assisted sigmoidectomy for diverticular disease. Int J Colorectal Dis 27:781–787.

Rafferty J, Shellito P, Hyman NH, Buie WD. 2006. Practice Parameters for Sigmoid Diverticulitis. Dis Colon Rectum 49:939–944.

Ritz J-P, MD KSL, MD BF, PhD AS, FRCS HJBMF, MD CH. 2011. Outcome of patients with acute sigmoid diverticulitis: Multivariate analysis of risk factors for free perforation. Surgery 149:606–613.

Wasvary H, Turfah F, Kadro O, Beauregard W. 1999. Same hospitalization resection for acute diverticulitis. Am Surg 65:632–5– discussion 636.

**LASER trial. LAparoscopic elective Sigmoid resection following divERticulitis - A multicenter, prospective, randomized clinical trial.**

Suomenkielinen nimi: Laparoskooppinen elektiivinen sigmaresektio divertikuloosin vuoksi - randomoitu prospektiivinen monikeskustutimus

Tutkimussuunnitelma v.1.7 (11.1.2017) (Changes in GREEN)

Alex Santos[1], Panu Mentula[1], Suvi Rasilainen[2], Tero Rautio[3], Arto Turunen[4], Samuli Aho[5], Tarja Pinta[6], Mikael Victorzon[7], Jukka Karvonen[8], Mirjami Uotila-Nieminen[9], Eero Kangas[10], Juha Rinne[11], Anna Fagerström[12], Aleksi Lähdesmäki[13], Tom Scheinin[1], Ville Sallinen[1]; LASER trial study group

LASER trial study group members: Matti Kairaluoma[5], Anu Erlich[5], Risto Juusela[7], Jan Andersén[7], Hillar Jääger[7], Heini Savolainen[12], Selja Koskensalo[1], Olli Kruuna[1], Heikki Huhtinen[8], Pirita Varpe[8], Jussi Luhtala[13]

[1] Helsingin yliopistollinen keskussairaala, Kirurginen sairaala
[2] Helsingin yliopistollinen keskussairaala, Jorvin sairaala
[3] Oulun yliopistollinen sairaala
[4] Kanta-Hämeen keskussairaala
[5] Keski-Suomen keskussairaala
[6] Etelä-Pohjanmaan keskussairaala
[7] Vaasan keskussairaala
[8] Turun yliopistollinen keskussairaala
[9] Pohjois-Karjalan keskussairaala
[10] Etelä-Karjalan keskussairaala
[11] Päijät-Hämeen keskussairaala
[12] Kuopion yliopistollinen sairaala
[13] Hyvinkään sairaala

## Tausta

Divertikuloosi on yhä yleisempi vaiva ikääntyvässä väestössä. Arviolta 25% sairastuu akuutiin divertikuliittiin (Biondo et al., 2012). Akuutin divertikuliitin prognoosi on sidoksissa sen vaikeusasteeseen Hinchey-luokituksen perusteella. Lievimmät divertikuliitit paranevat itsestään, kun taas mortaliteetti fekaalisen peritoniitin aiheuttaneeseen divertikuliittiin on korkea (Wasvary et al., 1999). Vatsakalvontulehduksen aiheuttaneet divertikuliitit leikataan yleensä päivystyksenä tehtävällä sigmaresektiolla ja mahdollisella suolen sisällön diversiolla avanteen kautta. Sen sijaan lievemmät akuutin divertikuliitin muodot hoidetaan yleensä konservatiivisesti suonensisäisellä antibiootilla ja absessit dreeneraten.

Aiemmin on suositeltu, että potilaille, joilla on toistuvia divertikuliitteja, tehtäisiin elektiivinen sigmaresektio, jotta voidaan jatkossa välttää komplisoituneen divertikuliitin ilmentyminen (Rafferty et al., 2006). Kuitenkin retrospektiivisissä töissä on todettu, että puhjennut divertikulitti on useimmiten ensimmäinen, ja komplisoituneen divertikuliitin ilmeneminen vähenee divertikuliittien uusiessa (Chapman et al., 2006; Ritz et al., 2011). Toisekseen on suositeltu komplisoituneen divertikuliitin (esim. paise) leikkaamista elektiivisesti (Rafferty et al., 2006). Myös tämä suositus on hiljattain kuitenkin

kyseenalaistettu (Gaertner et al., 2013). Toisaalta on osoitettu, että elektiivinen sigmaresektio parantaa elämänlaatua valikoiduilla potilailla, jotka ovat sairastaneet joko toistuvia divertikuliitteja tai komplisoituneen divertikuliitin (Forgione et al., 2009; Pasternak et al., 2011).

Tämänhetkinen tieto siitä kenelle elektiivinen sigmaresektio tulisi tehdä on erittäin rajoittunutta, ja perustuu retrospektiivisiin vertaileviin sarjoihin, tai prospektiivisiin tutkimuksiin, joista puuttuu verrokkiryhmä. Tällä hetkellä ei ole julkaistu yhtään randomoitua, prospektiivista tutkimusta, jossa verrattaisiin leikkaushoitoa konservatiiviseen hoitoon potilailla, joilla on relatiivinen indikaatio (toistuvat divertikuliitit, komplisoitunut divertikuliitti, kiputila akuutin divertikuliitin jälkeen) elektiiviseen sigmaresektioon. Laajassa tuoreessa divertikuliitin hoitosuosituksessa ei voida antaa definitiivisiä suosituksia potilaiden valikoimiseen elektiiviseen sigmaresektioon randomisoitujen töiden puutteen vuoksi (Andersen et al., 2012).

Tutkimuksen päätarkoituksena on selvittää elektiivisen sigmaresektion vaikutusta gastroenterologiseen elämänlaatuun (Gastrointestinal Quality of Life) vertailemalla leikkaushoitoa konservatiiviseen hoitoon randomoidussa prospektiivisessa monikeskustutkimuksessa. Toissijaisena tarkoituksena on selvittää divertikuliittien uusiutumista, kuolleisuutta sekä leikkauskomplikaatioita.

## Hypoteesi

Elektiivinen sigmaresektio parantaa elämänlaatua ja vähentää divertikuliittien uusiutumista seurannassa.

## Menetelmät

### Tutkimuspaikat

Helsingin yliopistollinen keskussairaala, Kirurginen sairaala
Helsingin yliopistollinen keskussairaala, Jorvin sairaala
Oulun yliopistollinen sairaala
Kanta-Hämeen keskussairaala
Keski-Suomen keskussairaala
Etelä-Pohjanmaan keskussairaala
Vaasan keskussairaala
Turun yliopistollinen keskussairaala
Pohjois-Karjalan keskussairaala
Etelä-Karjalan keskussairaala
Päijät-Hämeen keskussairaala
Kuopion yliopistollinen sairaala
Hyvinkään sairaala

### Interventioryhmät

3. Elektiivinen sigmaresektio
4. Konservatiivinen hoito (elämäntapaohjeet, kuitulisä)

### Tutkimuspotilaat

Inkluusiokriteerit

4. Toistuvat vas. puoleiset koolon divertikuliitit (vähintään 3, vähintään 2v sisällä, ainakin yksi TT:llä varmistettu)

5. Aiemmin sairastettu komplisoitunut divertikuliitti* (poislukien divertikuliitit, joissa vain perikoolista ilmaa)
6. TT:llä varmistetun vas. puoleisen koolon divertikuliitin jälkeiset kivut tai suolen toiminnan vaikeudet, jotka jatkuneet yli 3kk akuutin hoidon jälkeen

   * Komplisoituneella divertikuliitilla tarkoitetaan tietokonetomografialla verifioitua akuuttia vas. puoleista koolon divertikuliittia, johon liittyy fisteli, striktuura, absessi tai suolen ulkopuolella oleva tietokonetomografialla todettu ilma.

Ekskluusiokriteerit

12. Multimorbiditeetti, joka estää elektiivisen leikkausharkinnan
13. Este laparoskopialle (esim. anestesiologinen syy)
14. Koolonin striktuura
15. Fisteli (kolokutaani, kolovesikaali, kolovaginaali, tms)
16. Aktiivinen syöpä
17. Aiempi vasemman puoleisen koolonin tai rektumin resektio
18. Akuutti divertikuliitti, joka ei ole rauhoittunut (tulehdusarvot normaalistuneet, kuumeilu loppunut)
19. Kolonoskopiaa/sigmoideoskopiaa tai paksusuolen-tietokonetomografiaa (virtuaalikolonoskopiaa) ei ole tehty 2v sisällä randomisaatiosta
20. Ikä < 18 tai > 75 vuotta
21. Raskaus
22. Kyvyttömyys vastata elämänlaatukyselyihin (esim. dementia, psykiatrinen sairaus,tms)

**Satunnaistaminen**

Potilasta satunnaistetaan 1:1 joko elektiiviseen sigmaresektioon tai konservatiiviseen hoitoon. Potilaat satunnaistetaan stratifioidusti inkluusiokriteereiden perusteella, siten että molemmissa randomisaatioryhmissä olisi yhtä monta potilasta kustakin inkluusiokriteeriryhmästä. Satunnaistaminen tehdään tietokoneen avulla keskitetysti HYKS:stä käsin. Käytännössä randomisaation tekevä henkilö ottaa internetin välityksellä yhteyden HYKS:n palvelimeen, joka antaa tutkijalle tiedon potilaan randomisaatioryhmästä. Randomisaatio tehdään poliklinikkakäynnin yhteydessä, kun potilas on ensin antanut kirjallisen suostumuksen tutkimukseen osallistumiseen. Ryhmän perusteella valitaan hoito, eli potilas joko asetetaan leikkausjonoon elektiivista sigmaresektiota varten tai hänelle annetaan elämäntapaohjeistus sekä ohjeistus käyttää kuitulisää. Randomisaation yhteydessä potilas täyttää SF-36 ja GIQLI elämänlaatukyselykaavakkeet. Potilaat analysoidaan intention-to-treat periaatteen mukaisesti alkuperäisen randomisaatioryhmän mukaan.

**Interventio**

2. Elektiivinen laparoskooppinen sigmaresektio

   Potilas asetetaan leikkausjonoon elektiivistä laparoskooppista sigmaresektiota varten. Leikkaus suoritetaan **maksimissaan 3 kuukauden sisällä** randomisaatiosta. CRF #3 täytetään juuri ennen leikkausta (esim. preoperatiivisen

käynnin yhteydessä). CRF #2 täytetään potilaan kotiutuessa elektiivisen sigmaresektion jälkeen. Leikkauksen jälkeen potilaalle annetaan elämäntapaohjeistus sekä suositus kuitulisän käytöstä.

Tekniikka laparoskopia

7. Porttien asetus vapaa
8. Distaalinen katkaisukohta ylärektum, promontoriumin alapuolella
9. Proksimaalinen katkaisukohta oraalisessa sigmassa tai laskevassa koolonissa
10. Flexura lienalis mobilisoidaan tarvittaessa, jotta saadaan kiristyksetön sauma
11. Verisuonitus pyritään jättämään mahdollisimman hyväksi. A. mesenterica inferior ja a. rectalis superior pyritään säästämään. Tarvittaessa verisuonia voidaan kuitenkin katkaista esimerkiksi kiristyksettömän sauman aikaansaamiseksi tai mikäli dissektio-olosuhteeet ovat vaikeat.
12. Suolianastomoosi ILS-laitteella

3. Konservatiivinen hoito

Elämäntapaohjeistus kirjallisena

- Lääkärikirja Duodecim Tietoa potilaalle: Suolen umpipussi tauti, 12.11.2012 Pertti Mustajoki (www.terveysportti.fi)
- Lääkärikirja Duodecim Tietoa potilaalle: Ummetus 8.10.2012 Pertti Mustajoki (www.terveysportti.fi)

Suullinen suositus kuitulisän käytöstä.

Toiminta jouduttaessa elektiiviseen sigmaresektioon potilaalla, joka on randomoitu konservatiivisen hoidon ryhmään:

Konservatiivista hoitoa tulisi kokeilla vähintään 6kk randomisaatiosta (=ensimmäiseen elämänlaatukyselyyn saakka) ennen kuin voidaan päättää, että se on epäonnistunut ja potilas ohjata elektiiviseen sigmaresektioon. Luonnollisesti operatiivisen hoidon voi suorittaa aikaisemminkin, mikäli potilaalle on kehittynyt absoluuttisia indikaatioita elektiiviseen sigmaresektioon (esim. fisteli, striktuura). Mikäli potilas, joka on randomoitu konservatiiviseen hoitoon, joudutaan hoitamaan elektiivisella sigmaresektiolla, tulee potilaasta täyttää leikkauspäätöksen hetkellä GIQLI ja SF-36 elämänlaatukyselyt. Leikkauksen jälkeen potilasta seurataan alkuperäisen seurantascheeman mukaisesti (eli kyselyt juuri ennen leikkausta (preoperatiivinen käynti, tms.), 6kk, 12kk, 24kk, 48kk ja 96kk päästä **alkuperäisestä randomisaatiosta**). Normaalien tutkimuseettisten periaatteiden mukaisesti potilas voi myös itse jättäytyä kokonaan pois tutkimuksesta omasta halustaan, jolloin hänen seurantansa loppuu ja hänet hoidetaan normaalien hoitolinjojen ja hoitavan lääkärin harkinnan mukaisesti.

**Tutkittavat muuttujat**

Päävastemuuttuja

Gastrointestinal Quality of Life Index (GIQLI) - ero 0kk ja 6kk kohdalla randomisaatiosta

Toissijaiset muuttujat

10. Gastrointestinal Quality of Life Index (GIQLI) 12kk, 24kk, 48kk ja 96kk kohdalla randomisaatiosta
11. SF-36 elämänlaatukyselyn pistemäärä 6kk, 12kk, 24kk, 48kk ja 96kk kohdalla randomisaatiosta
12. Divertikuliittien uusiutuminen ja Hinchey-luokka
13. Joutuminen päivystykselliseen leikkaukseen divertikuloosin/divertikuliitin vuoksi
14. Joutuminen elektiiviseen sigmaresektioon (konservatiivinen hoitoryhmä)
15. Elektiivisesta sigmaresektiosta aiheutuneet komplikaatiot
16. Mortaliteetti (mistä tahansa syystä molemmissa ryhmissä)
17. Divertikuloosin komplikaatiot (fisteli, striktuura)
18. Avanne (pysyvä / väliaikainen)

**Seuranta**

Liitteenä olevasta vuokaaviosta käy ilmi potilaiden randomisaatio, hoitotoimenpiteiden ajoittaminen sekä seuranta.
Potilaille varataan soittoaika 6kk päähän randomisaatiosta, jossa tiedustellaan vointi, ja voidaan päättää mahdollisesta hoitotavan muutoksesta (konservatiivisen hoidon ryhmän potilas ohjata elektiiviseen sigmaresektioon).
Potilaita seurataan lisäksi postitse lähetettävin kyselykaavakkein (SF-36, GIQLI, sekä tutkijoiden laatima kyselykaavake, kts. liite) juuri ennen leikkausta (preoperatiivinen käynti, tms.) sekä 6kk, 12kk, 24kk, 48kk ja 96kk päästä randomisaatiosta. Samat kyselykaavakkeet täytetään myös juuri ennen elektiivistä sigmaresektiota. Mikäli kyselykaavakkeen vastauksissa on epäselvyyttä, tutkija ottaa tarvittaessa potilaaseen yhteyttä puhelimitse selvittääkseen epäselvyydet.
Kunkin keskuksen tutkija kerää oman keskuksensa kyselykaavakkeen ja tarkistaa tässä yhteydessä myös oman alueensa potilasasiakirjoista merkinnät. Näiden perusteella tutkija täyttää CRF #3-lomakkeen, joka täytetään siis jokaisessa seuranta-aikapisteessä.

**Kustannukset**

Tutkimuksen ei arvioida aiheuttavan merkittäviä ylimääräisiä kuluja, sillä elektiivinen sigmaresektio on normaalia hoitoa jo tällä hetkellä ja inkluusiokriteerit noudattavat yleisiä hoitosuosituksia. Ainoat kustannukset tulevat potilaalle tehtävästä puhelinsoitosta (6kk kohdalla) sekä kyselykaavakkeiden postituskustannuksista. Tutkimus saattaa jopa säästää sairaaloiden kuluja, sillä puolet potilaista randomoidaan konservatiiviseen hoitoon ja heidän osaltaan leikkauskustannukset puuttuvat.

**Kerättävät parametrit**

Case Report Form (kts. liite). Case report form #1 täytetään randomisaation yhteydessä (poliklinikkakäynti). Case report from #2 täytetään elektiivisesti leikatun potilaan

kotiutuessa. Case report form #3 täytetään jokaisessa seuranta-aikapisteessä (juuri ennen leikkausta (preoperatiivinen käynti, tms.), 6kk, 12kk, 24kk, 48kk, 96kk).

**Otoskoko**
Aikaisemmissa julkaisuissa preoperatiivinen GIQLI on ollut 95-100 (SD 22) ja postoperatiivinen GIQLI 112-114 (SD 19-22) (Forgione et al., 2009; Pasternak et al., 2011). Otoskokolaskelma tehtiin käytäen näitä arvoja, ja tutkimuksessa pyritään näyttämään 12 GIQLI-pisteen ero, standardideviaatioksi oleteteaan molemmissa ryhmissä 22 pistettä. Käyttäen 0.05 alfaa, 0.9 voimaa ja 1:1 allokaatiota saadaan otoskooksi per ryhmä 60 potilasta. Otoskokolaskema tehtiin käyttäen G*Power 3.1.5.1-ohjelmaa (comparison of means, two-tailed, A priori). Seurannassa arvioidaan häviävän noin 10% potilaista, joten lopulliseksi otoskooksi tulee yhteensä 133 potilasta.

**Aikataulu, välianalyysi ja tutkimuksen päättyminen**
Tutkimus suunnitellaan alkavan kesällä/syksyllä 2014. Tutkimuksen puolivälissä eli 66 potilaan kohdalla suoritetaan välianalyysi. Mikäli jo tässä vaiheessa saadaan päävastemuuttujaan tilastollisesti merkittävä ero, ei tutkimusta jatketa eteenpäin. Muuten tutkimus jatkuu, kunnes 133 potilasta on randomoitu. Potilaiden rekrytoimisen arvioidaan kestävän n. viisi vuotta. Seurantavaihe kestää tästä vielä kahdeksan vuotta eteenpäin.

**Rekisteröinti**
Tutkimus rekisteröidään ennen satunnaistamisten aloittamista clinicaltrials.gov-palveluun.

**Tietosuoja**
Randomoitujen potilaiden henkilötiedot kerätään tutkimuskansioon, sekä heistä täytetään CRF #1, #2 ja #3. Potilaista, jotka täyttävät inkluusiokriteerit ja siten arvioidaan tutkimukseen osallistumista, pidetään kirjanpitoa, siitä kuinka monta potilasta jää pois tutkimuksesta, ja mistä syystä (ekskluusiokriteerin täyttyminen, kieltäytyminen, tms). Tutkimuksesta poisjääneiden potilaiden henkilötietoja ei kerätä. Kerättävä aineisto säilytetään lukitussa tilassa ja sähköiset tiedot tietokoneilla salasanalla ja salauksella suojattuina.

**Viitteet**

Andersen JC, Bundgaard L, Elbrønd H, Laurberg S, Walker LR, Støvring J, Danish Surgical Society. 2012. Danish national guidelines for treatment of diverticular disease. Dan Med J 59:C4453.

Biondo S, Lopez Borao J, Millan M, Kreisler E, Jaurrieta E. 2012. Current status of the treatment of acute colonic diverticulitis: a systematic review. Colorectal Disease 14:e1–e11.

Chapman JR, Dozois EJ, Wolff BG, Gullerud RE, Larson DR. 2006. Diverticulitis: a progressive disease? Do multiple recurrences predict less favorable outcomes? Ann Surg 243:876–830; discussion 880–3.

Forgione A, Leroy J, Cahill RA, Bailey C, Simone M, Mutter D, Marescaux J. 2009. Prospective Evaluation of Functional Outcome After Laparoscopic Sigmoid Colectomy. Ann Surg 249:218–224.

Gaertner WB, Willis DJ, Madoff RD, Rothenberger DA, Kwaan MR, Belzer GE, Melton GB. 2013. Percutaneous Drainage of Colonic Diverticular Abscess. Dis Colon Rectum 56:622–626.

Pasternak I, Wiedemann N, Basilicata G, Melcher GA. 2011. Gastrointestinal quality of life after laparoscopic-assisted sigmoidectomy for diverticular disease. Int J Colorectal Dis 27:781–787.

Rafferty J, Shellito P, Hyman NH, Buie WD. 2006. Practice Parameters for Sigmoid Diverticulitis. Dis Colon Rectum 49:939–944.

Ritz J-P, MD KSL, MD BF, PhD AS, FRCS HJBMF, MD CH. 2011. Outcome of patients with acute sigmoid diverticulitis: Multivariate analysis of risk factors for free perforation. Surgery 149:606–613.

Wasvary H, Turfah F, Kadro O, Beauregard W. 1999. Same hospitalization resection for acute diverticulitis. Am Surg 65:632–5– discussion 636.
