# Supplementary material for: Comparing Laparoscopic Elective Sigmoid Resection With Conservative Treatment in Improving Quality of Life of Patients With Diverticulitis: The Laparoscopic Elective Sigmoid Resection Following Diverticulitis (LASER) Randomized Clinical Trial
Source: JAMA Surg. 2020 Nov 18;156(2):1–9. doi: 10.1001/jamasurg.2020.5151 (PMC7675217; doi:10.1001/jamasurg.2020.5151)
Supplement: Supplement 2. — eAppendix 1. Suolen umpipussitauti (divertikuloosi ja divertikuliitti). eAppendix 2. Diverticular disease (diverticulosis and diverticulitis). eAppendix 3. Ummetus. eAppendix 4. Constipation. [file jamasurg-e205151-s002.pdf]

Supplementary Online Content

Santos A, Mentula P, Pinta T, et al. Comparing laparoscopic elective sigmoid resection with conservative treatment in improving quality of life of patients with diverticulitis: the Laparoscopic Elective Sigmoid Resection Following Diverticulitis (LASER) Randomized Clinical Trial. *JAMA Surg*. Published online November 18, 2020. doi:10.1001/jamasurg.2020.5151

eAppendix 1. Suolen umpipussitauti (divertikuloosi ja divertikuliitti)
eAppendix 2. Diverticular disease (diverticulosis and diverticulitis)
eAppendix 3. Ummetus
eAppendix 4. Constipation

This supplementary material has been provided by the authors to give readers additional information about their work.

© 2020 Santos A et al. JAMA Surgery.

eAppendix 1. Suolen umpipussitauti (divertikuloosi ja divertikuliitti)

Lääkärikirja Duodecim
12.11.2012
sisätautien erikoislääkäri Pertti Mustajoki

» Oireet
» Itsehoito
» Hoito
» Ehkäisy

Suolen umpipussitaudissa eli divertikuloosissa paksusuolen seinämään on syntynyt pullistumia eli divertikkeleitä (ks. röntgenkuva 1). Ne syntyvät hiljalleen vuosien kuluessa ilmeisesti siksi, että suolen sisällä oleva paine pullistaa suolen heikkoa kohtaa ulospäin. Ummetustaipumus ja vähäkuituinen ruoka lisäävät painetta paksusuolessa ja siten myös umpipussien muodostumista.

Pullistumia syntyy ikääntymisen mittaan. Alle 45-vuotiailla suolen yksittäisiä pullistumia (divertikkeleitä) on noin kymmenellä sadasta, yli 80-vuotiailla suurimmalla osalla. Silloinkun pullistumia syntyy useita, yleensä useita kymmeniä, puhutaan umpipussitaudista eli divertikuloosista.

Oireet

Umpipussitauti ei tunnu lainkaan, ja useimmilla ihmisillä se on koko elämän täysin oireeton. Noin joka viidennellä umpipussitautiin liittyy oireita. Tavallisesti ne ilmenevät erilaisina "ruuansulatushäiriöinä": epäsäännöllinen vatsan toiminta, ummetus, ilmavaivat. Oireet ovat usein samantyyppisiä kuin ärtyvässä suolessa (ks. 1).

Sadasta umpipussitaudista kärsivästä 1–2:lle kehittyy äkillinen umpipussien tulehdus eli divertikuliitti. Tällöin esiintyy yleensä vasemmalla alavatsalla päiväkausia kestävää kipua ja lievää kuumetta.

Joskus harvoin umpipussi saattaa vuotaa verta suolen sisään, jolloin voi syntyä anemiaa. Pahin mutta onneksi hyvin harvinainen lisätauti on umpipussin puhkeaminen, johon liittyy äkisti alkava kova vatsakipu. Seurauksena on pikaista hoitoa vaativa vatsakalvon tulehdus.

Itsehoito

Jo syntyneet suolen umpipussit eivät palaudu, mutta tulehdusta ja muita lisätauteja voidaan todennäköisesti ehkäistä ummetuksen (ks. 2) tehokkaalla hoidolla. Tähän kuuluu kuitupitoinen ruokavalio, jota on selostettu ummetuksen hoidon yhteydessä.

Hoito

Paksusuolen umpipusseja todetaan usein, kun muiden oireiden vuoksi tehdään paksusuolen tähystys tai varjoainetutkimus. Näin todettujen oireettomien umpipussien vuoksi ei tarvitse mennä lääkäriin, mutta ummetuksen itsehoito ja kuitupitoinen ruokavalio ovat paikallaan.

© 2020 Santos A et al. JAMA Surgery.

Jos on todettu suolen umpipusseja ja ilmaantuu voimakkaita vatsakipuja (ks. 3), on syytä hakeutua viivyttelemättä hoitoon. Siinä vaiheessa yleensä ei tiedetä, johtuvatko oireet umpipussien lisätaudeista vai jostain muusta vatsan alueen sairaudesta. Lääkärin tutkimuksessa oireiden syy selviää.

Umpipussien tulehdusta hoidetaan suonensisäisillä antibiooteilla sairaalassa. Tulehduksen vuoksi leikkaushoitoa ei yleensä tarvita. Ainoastaan jos tulehduksia esiintyy toistuvasti, harkitaan umpipusseja sisältävän suolen osan poistamista leikkauksella. Puhjennut umpipussi vaatii välittömän leikkauksen. Myös verta vuotava umpipussi joudutaan yleensä leikkaamaan.

Ehkäisy

Paksusuolen umpipussien syntyä voidaan ehkäistä samoilla toimenpiteillä kuin ummetusta eli kuitupitoisella ruokavaliolla ja liikunnalla, ks. 2 .

Käytettyjä lähteitä

Kellokumpu I. Divertikuliitti ja divertikuloosi. Lääkärin tietokannat/Lääkärin käsikirja [online]. Päivitetty 17.4.2009. Helsinki: Kustannus Oy Duodecim.

Mäkelä J ym. Koolonin divertikuliitin hoitolinjat. Duodecim 2010;126:788-93. (Lue artikkeli)

Artikkelin tunnus: dlk00094 (005.209)
© 2020 Kustannus Oy Duodecim

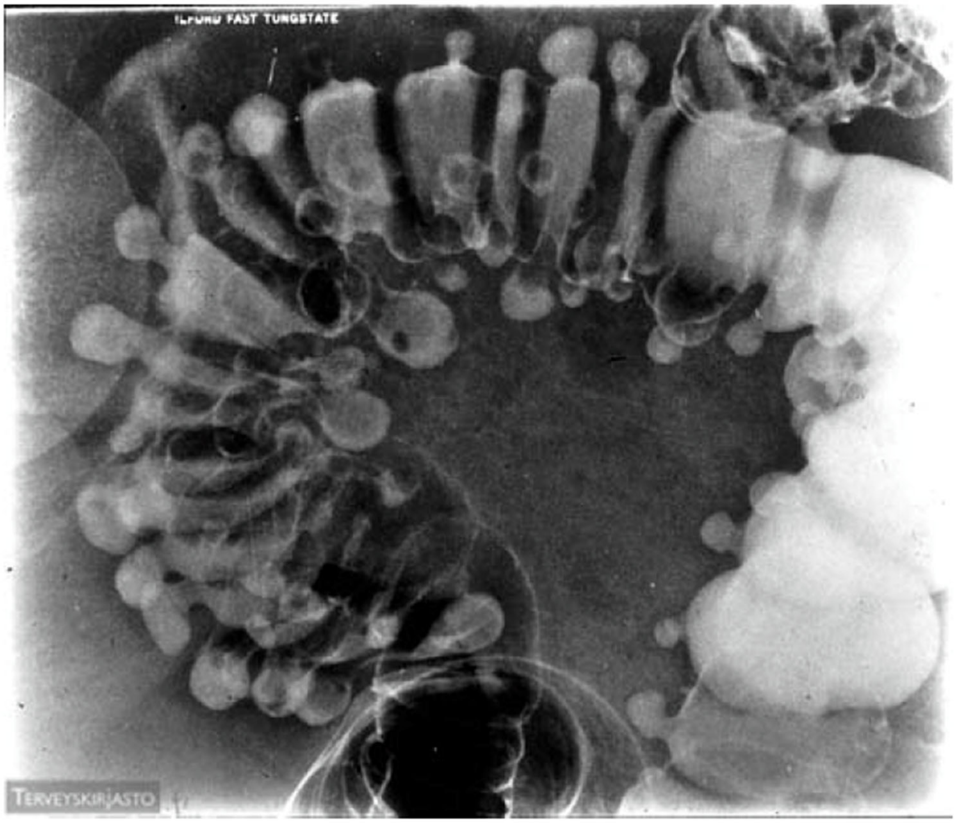

Suolen umpipussitauti (divertikuloosi). Varjoaineröntgenkuva paksusuolesta, jossa näkyy divertikkeleitä eli suolen umpipusseja (vaaleat palleromaiset muodostumat suolen reunamilla). Lisää tietoa divertikuloosista, ks. ? .

© 2020 Santos A et al. JAMA Surgery.

eAppendix 2. Diverticular disease (diverticulosis and diverticulitis)

Lääkärikirja Duodecim

12.11.2012

Specialist in internal medicine, Pertti Mustajoki

>> Symptoms

>> Self treatment

>> Treatment

>> Prevention

Intestinal diverticulosis results from a bulging within the colon wall (see. The X-ray image 1). They are form gradually over the years, apparently because the pressure within the bowel puff bowel weak points outward. Constipation and low-fiber food increase the pressure in the colon and hence the formation of cachets.

These bulging areas appear and increase with age. Under 45 years old have isolated diverticula about 10% of the times. When age reaches 80, most patients will have diverticula. When there are many diverticula, often dozens of them, we call it diverticulosis.

Symptoms

Diverticular disease does have exterior signs and for most people it is asymptomatic throughout life. Approximately only one out of five cases are associated with symptoms. They are usually manifested as various "digestive disorders": irregular stomach function, constipation, flatulence. The symptoms are often similar irritable bowel syndrome.

One or two out of every 100 people with diverticular disease disease develops sudden sepsis or diverticulitis. This usually results in pain and mild fever for days in the lower left abdomen.

On rare occasions, can happen diverticular bleeding in the gut, which may result in anemia. The worst but fortunately rare disease is the bursting of a closed pouch with sudden onset of severe stomach pain. The result is an inflammation of the peritoneum that requires urgent treatment.

© 2020 Santos A et al. JAMA Surgery.

Home treatment

Once diverticulosis develops, it won't disappear but the inflammation and other morbidities may be more likely to be prevented with effective treatment of constipation. This includes a high-fiber diet that has been described in the treatment of constipation.

Treatment

Colonic diverticulitis is often found when a colon endoscopy or contrast agent research is done usually for other reasons. These asymptomatic closed pouches require no further medical intervention other than self-treatment for constipation and a fiber-rich diet.

If you have been diagnosed with intestinal diverticulosis and experience strong abdominal pain, it is advisable to seek treatment immediately. At this stage, it is generally not known whether the symptoms are related to diverticulosis or any other abdominal disorder. In a doctor's examination, the cause of the symptoms will be clear.

Inflammation of the diverticula are treated with intravenous antibiotics in an hospital setting. Surgery is usually not needed because of diverticula inflammation. Only if the inflammation episodes tend to happen frequently one might consider surgery for the removal of affected bowel. A perforated diverticula requires immediate surgery. Surgery is also often required if diverticula bleeds.

Rarely, the diverticula bursts and requires immediate surgery. Another very rare disorder is bleeding, which sometimes requires surgery.

Prevention

Colonic diverticulosis can be reduced by the same measures as constipation: fiber-containing diet and exercise.

Sources

Kellokumpu I. Divertikuliitti ja divertikuloosi. Lääkärin tietokannat / Lääkärin. Kustannus Oy Duodecim.

© 2020 Santos A et al. JAMA Surgery.

Mäkelä J ym. Koolonin divertikuliitin hoitolinjat. Duodecim 2010;126:788-93

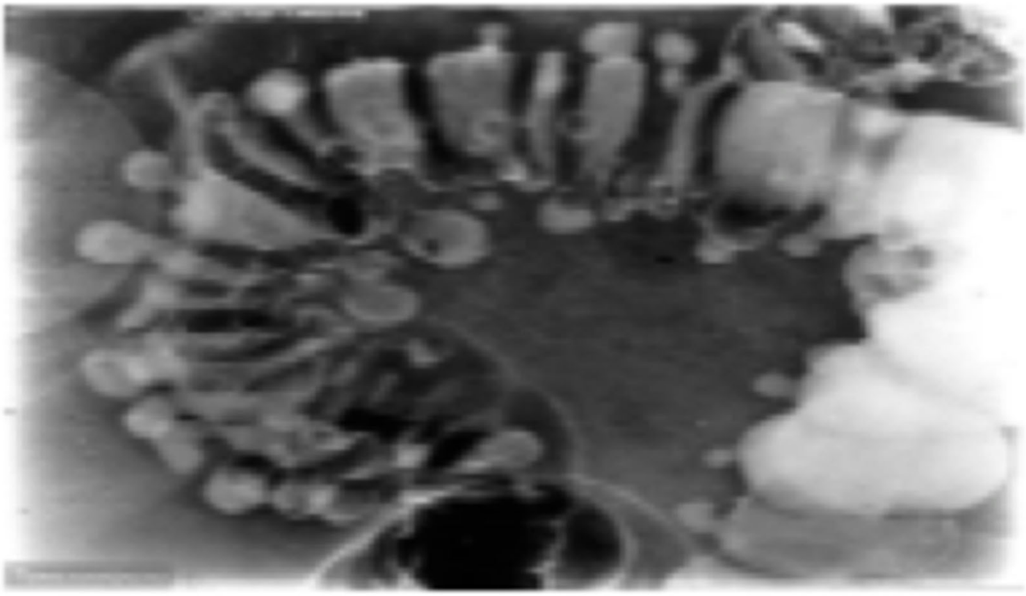

Diverticulosis. Contrast X-ray image of colon, which shows diverticula (light ball-shaped formation in next to the bowel).

© 2020 Santos A et al. JAMA Surgery.

eAppendix 3. Ummetus

Lääkärikirja Duodecim
8.10.2012
sisätautien erikoislääkäri Pertti Mustajoki

» Ummetuksen syyt
» Ummetuksen itsehoito
» Kuitupitoinen ruokavalio
» Liikunta
» Säännöllinen ulostamisrytmi
» Apteekkilääkkeiden käyttö ummetukseen
» Milloin hoitoon?
» Ummetuksen hoito
» Ummetuksen ehkäisy

Ihmisen suoli tyhjenee normaalisti 8–72 tunnin välein. Ummetus on kyseessä, kun suoli tyhjenee harvemmin kuin kolme kertaa viikossa tai jos ulostus on niin kiinteää, että ulostaminen on useimpina kertoina vaikeaa. Terveistä aikuisista ummetusta esiintyy 1–6:lla sadasta. Ummetusta on enemmän iäkkäillä, vähän liikkuvista vanhuksista, joista jopa 8 kymmenestä kärsii ummetuksesta.

Ummetuksen syyt

Elämäntavat vaikuttavat ratkaisevasti suolen tyhjenemiseen. Ummetusta aiheuttavat vähäinen liikunta, ruoan vähäinen kuitupitoisuus ja ilmeisesti myös liian vähäinen nesteiden saanti. Toistuva ulostuksen pidättäminen saattaa myös johtaa ummetukseen siksi, että suoli "oppii" tyhjenemään harvoin.

Monet lääkkeet aiheuttavat ummetusta. Voimakkaat kipulääkkeet opiaatit kovettavat säännöllisesti vatsaa. Muita samantyyppisesti vaikuttavia lääkkeitä ovat verapamiili, monet psyyken lääkkeet, eräät Parkinsonin taudin lääkkeet, rautavalmisteet ja nesteenpoistolääkkeet (diureetit).

Eräiden aineenvaihduntasairauksien, esimerkiksi kilpirauhasen vajaatoiminnan, yhtenä oireena voi esiintyä ummetusta. Joissakin psyyken häiriöissä, kuten anoreksia nervosassa ja masennuksessa, vatsan toiminta on hidastunut. Ummetus on yksi oire toiminnallisissa vatsavaivoissa (ks. 1).

Ummetuksen itsehoito

Ummetuksen hoidon perusta ovat sopivat muutokset ruokavaliossa, riittävä liikunta ja säännöllinen ulostamisrytmi. Lääkkeet ja muut toimenpiteet ovat aina toissijaisia hoitomuotoja. Jokaisen ummetuspotilaan tulisi pyrkiä vakavissaan toteuttamaan alla esitettyjä itsehoidon ohjeita. Ne eivät vaikuta välittömästi, vaikutus voidaan arvioida vasta 1 - 2 kuukauden kuluttua. Muutoksien tulee olla pysyviä, jotta niiden ummetusta ehkäisevä teho säilyisi.

Kuitupitoinen ruokavalio

Ravinnon kuidut vähentävät ummetusta, koska ne lisäävät suolen sisältöä sekä sitovat suolessa vettä ja siten kuohkeuttavat ulostetta. Kuituja voi lisätä seuraavasti:

© 2020 Santos A et al. JAMA Surgery.

Kaikissa kasvikunnan tuotteissa, viljassa, kasviksissa, hedelmissä ja marjoissa, on kuituja. Tehokkaimmin suolen sisältöä lisää viljan kuitu, jota on täysjyväleivissä ja -puuroissa. Keskimäärin suomalaiset saavat ravinnosta runsas 20 grammaa kuituja. Ummetuspotilaan pitää saada ruuasta vähintään 30–35 grammaa kuitua päivässä. Usein ummetuksen hoitamisen kuituja tarvitaan vielä enemmän.

Ylimääräisiä kuituja saa vehnä- tai ruisleseistä tai pellavansiemenrouheista. Kuitulisä voi aiheuttaa aluksi väliaikaisesti kaasunlisäystä ja vatsavaivoja, minkä vuoksi leseen käyttö kannattaa aloittaa vähitellen. Aluksi 1–2 teelusikallista päivässä, minkä jälkeen annosta lisätään päivittäin, kunnes lesemäärä on 3–6 ruokalusikallista päivässä. Leseen voi liuottaa veteen tai sekoittaa muuhun ruokaan. Ks. myös 100 kysymystä ravinnosta -kirjan artikkelit 2 ja 3 .

Apteekista saa ummetusta varten suunniteltuja erilaisia kuituvalmisteita. Apteekkihenkilökunta neuvoo niiden hankinnassa ja käytössä.

Luumut ja viikunat sisältävät kuitujen lisäksi suolen toimintaa lisääviä aineita. Sopiva annos on 6–7 vedessä liotettua luumua liotusvesineen aamulla.

Nestettä juodaan runsaasti, vähintään kaksi litraa päivässä, etenkin jos käyttää kuitulisänä leseitä tai apteekkivalmisteita.

Liikunta

Jos ihminen on laiska, suolikin toimii laiskasti. Siksi liikunnan lisääminen lieventää ummetusta. Liikunnan laadulla ei väliä, esimerkiksi reipas kävely sopii mainiosti joko erillisinä kävelylenkkeinä tai pienempinä annoksina hyötyliikuntana.

Säännöllinen ulostamisrytmi

Suolta voidaan opettaa säännöllisemmäksi: Aamulla juodaan lasi vettä (luumut voivat olla mukana) ja aamupalan jälkeen käydään "istunnolla". Harjoittelua jatketaan vähintään useita viikkoja, vaikka aluksi ulostusta ei tulisikaan.

Hyvä ulostamisasento auttaa peräsuolen tyhjenemistä. Paras asento on silloin, kun pöntöllä istuessa polvet ovat lonkkien tasoa korkeammalla. Suomalainen WC-istuin on tähän yleensä liian korkea. Silloin voi käyttää pallia jalkojen alla.

Apteekkilääkkeiden käyttö ummetukseen

Apteekista saa ilman reseptiä useilla eri tavoilla vaikuttavia ummetuslääkkeitä. Niitä annostellaan eri tavoin, pakkausselosteessa annettuja ohjeita pitää noudattaa.

Apteekista saa erilaisia kuituja sisältäviä suolen sisältöä lisääviä ummetuslääkkeitä, joita voi vaaratta käyttää pitkiäkin aikoja.

Laktiloli, laktuloosi ja makrogoli ovat paksusuolen nestettä lisääviä lääkkeitä, joita voi käyttää sitkeään ummetukseen. Vaikutus alkaa yleensä muutaman tunnin kuluttua mutta näkyy joskus vasta 1–2 päivän kuluttua. Näitä lääkkeitä ei ole tarkoitettu säännölliseen käyttöön, mutta hankalassa ummetuksessa niitä voi käyttää pitkiäkin aikoja.

Suolta stimuloivat lääkkeet sennosidi ja bisakodyyli on tarkoitettu vain tilapäiseen muutaman päivän käyttöön. Niitä on tabletteina, peräpuikkoina tai pienoisperäruiskeina. Näitä lääkkeitä voi vaaratta käyttää pahaan mahan

© 2020 Santos A et al. JAMA Surgery.

umpeen väliaikaisesti muutaman päivän. Pitkäaikaisessa käytössä suoli tottuu niihin, jonka jälkeen suoli ei enää toimi ilman lääkkeitä.

Milloin hoitoon?

- Jos itsehoidosta huolimatta ummetus häiritsee jokapäiväistä elämää.
- Jos ummetuksen lisäksi on muita oireita, esimerkiksi jatkuvaa väsymystä tai outoja vatsakipuja.
- Jos vuosia jatkunut suolen normaali toiminta muuttuu suhteellisen nopeasti (muutamassa viikossa) ummetukseksi.

Ummetuksen hoito

Lääkäri selvittää aluksi, onko ummetuksen syynä jokin muu sairaus tai käytetty lääke. Jos näitä ei löydy, voidaan ummetuksen lääkehoitoa tehostaa. Hankala ummetus voidaan laukaista peräruiskeella.

Lantionpohjan lihasten harjoittelu fysioterapeutin ohjauksessa auttaa tilanteessa, jossa alueen lihaksissa on kouristelutaipumusta (spastisuutta). Joskus itse ulostamistapahtumassa on häiriöitä, jolloin annetaan biopalautehoitoa. Siinä peräsuoleen asetetun pallon avulla harjoitellaan ulostamista ja samalla rekisteröidään lihaksiston toimintaa.

Ummetuksen ehkäisy

Ummetusta voidaan ehkäistä itsehoidon yhteydessä kerrotuilla keinoilla: liikkumalla säännöllisesti ja käyttämällä runsaasti kuitupitoisia kasviksia.

Käytettyjä lähteitä

Arkkila P. Aikuisen ummetus. Lääkärin tietokannat/Lääkärin käsikirja [online]. Helsinki: Päivitetty 28.4.2009. Kustannus Oy Duodecim.

Luukkonen P, Hillilä M. Vaikean ummetuksen hoito. Duodecim 2012;128:1869-76.

Artikkelin tunnus: dlk00092 (005.214)
© 2020 Kustannus Oy Duodecim

© 2020 Santos A et al. JAMA Surgery.

eAppendix 4. Constipation

Lääkärikirja Duodecim

8.10.2012

Specialist in internal Medicine, Pertti Mustajoki

>> Reasons for constipation

>> Home treatment

>> Fiber-rich diet

>> Exercise

>> Regular bowel movement

>> Pharmaceutical options

>> When to seek medical treatment

>> Treatment of constipation

>> Prevention of constipation

The emptying of the bowel varies individually between 8-72 hours on average. When the frequency of defecation is lower than three times a week or if the stools are so hard that defecation is frequently very difficult. In healthy adults difficult constipation happens in 1-6 out of 100. Constipation is more frequent in older patients, especially those who don't or can't move regularly.

Reasons for constipation

Lifestyle influences significantly bowel movements. Constipation can be caused by insufficient physical exercise, insufficient fiber intake and also dehydration. Unwillingness to go to the toilet as often as necessary also can cause constipation given that the bowel "learns" to function at a suboptimal rhythm.

Many different kinds of medication can cause constipation. Strong opioids make stool consistency significantly harder. Other drugs can cause similar effects namely verapamil, many different kind of psychiatric drugs, some Parkinson disease drugs, iron supplements and diuretics.

Some metabolic diseases like hypothyroidism can cause, among other things, constipation. In some psychiatric disorders like anorexia and depression, bowel movements significantly

© 2020 Santos A et al. JAMA Surgery.

decrease. Constipation can also be a common symptom in Irritable bowel syndrome.

Home treatment

The treatment of constipation is based upon dietary changes, physical exercise and frequent visits to the toilet. Medication and other procedures are always secondary options.

Each patient suffering from constipation should consider the following recommendations seriously. However, one must keep in mind that these do not work instantaneously. The efficiency of these measures can only be assessed after a period of 1-2 months. Also, these treatment norms should be maintained so that their effects might continue.

Fiber-rich diet

Dietary fiber improves constipations by means of increasing the content of stools inside the colon that binds to water and therefore make stool softer.

All vegetables contain some fiber. Different kinds of grain, salads, fruits and berries all include fiber. The higher fiber content can be found in integral grain and porridge. The average Finn gets on average 20gr of fiber from diet alone. Constipation patients should get at least 30-35gr daily and quite frequently even more.

Excess fiber is obtained from wheat or rye bran or linseed. Fiber supplementation may initially cause a temporary increase in gas and stomach problems, so it is advisable to gradually increment fiber intake. Initially 1 to 2 teaspoons a day, followed by a daily dose until the bran is 3 to 6 tablespoons a day. Fiber can be added to water or mixed with food. Please see also “100 questions about nutrition”-book chapters 2 and 3.

The pharmacy offers various fiber products designed for constipation. Pharmacy staff will advise on their purchase and use.

Plums and figs contain fibers and alsointestinal activity-enhancing agents. A suitable dose is 6-7 plums soaked in water in the morning with soaking water.

Drink plenty of fluid, at least two liters a day, especially if you use fiber or pharmacy supplements.

Exercise

If one lives a lazy life so will their bowels. That’s why by increasing exercise it is possible to relieve constipation. The quality of exercise does not matter, for example brisk walking is great either when walking alone for a longer period or in smaller doses during everyday life.

© 2020 Santos A et al. JAMA Surgery.

Regular bowel movement

Bowel movements can be taught to be more regular: In the morning, a glass of water (plums may be included) and after breakfast a visit to the toilet. This morning habit should be continued for at least several weeks even if you do not have defecation at first.

Good ergonomic position while defecating helps rectal emptying. The best position is when the knees are higher than the hips when sitting on the bench. The Finnish toilet is usually too high for this. Then you can use some support under your feet.

Pharmaceutical options

There are many different types of constipation medication that can be obtained without a prescription at the pharmacy. They are dosed in different ways, so it is paramount to follow the instructions in the package leaflet.

There is a range of fibers that can be used safely for long periods of time.

Lactulose and macrogol are drugs that enhance the liquid content inside the colon and may be used for persistent constipation. Effect begins to pick up usually within a few hours but usually only after one to two days. These drugs are not meant to be used on a regular basis, but can be used for long periods in case of severe constipation.

The gut-stimulating drugs sennoside and bisacodyl are for temporary use during a period of few days. They are available as tablets, suppositories, or miniature enemas. These medications can be safely used in severe cases for few days. During long-term use, the bowels become accustomed to them, after which the bowels will not function without medication.

When to seek medical treatment

- If, despite self-care, constipation disrupts everyday life.
- If you have other symptoms besides constipation, such as persistent tiredness or strange stomach pains.
- If the intestinal normal operation over many years is changing relatively quickly (a few weeks) into constipation.

Treatment of constipation

Your doctor will first determine if your constipation is due to another illness or medication. If these are not found, the treatment of constipation may be intensified. Difficult constipation can be treated with an enema.

Training the pelvic floor muscles under the guidance of a physiotherapist helps in the situation

© 2020 Santos A et al. JAMA Surgery.

where the muscles in the area have a tendency to spasm (spasticity). Occasionally, the defecation process itself is disturbed, in which case we advise bio-feedback therapy. In this kind of therapy a rectal ball is used to practice defecation while recording muscular activity.

Prevention of constipation

Constipation can be prevented by self-medication: regular exercise and high-fiber vegetables.

Sources

Arkkila P. Aikuisen ummetus. Lääkärin tietokannat / Lääkärin. Kustannus Oy Duodecim.

Luukkonen P, Hillilä M. Vaikean ummetuksen hoito. Duodecim 2012;128:1869-76.

Punkkinen J. Uusia lääkkeitä ummetuksen hoitoon. Suomen Lääkärilehti 2013;68:1113-1117.

© 2020 Santos A et al. JAMA Surgery.
