# Supplementary material for: Comparing Laparoscopic Elective Sigmoid Resection With Conservative Treatment in Improving Quality of Life of Patients With Diverticulitis: The Laparoscopic Elective Sigmoid Resection Following Diverticulitis (LASER) Randomized Clinical Trial
Source: JAMA Surg. 2020 Nov 18;156(2):1–9. doi: 10.1001/jamasurg.2020.5151 (PMC7675217; doi:10.1001/jamasurg.2020.5151)
Supplement: Supplement 3. — Data sharing statement. [file jamasurg-e205151-s003.pdf]

Data Sharing Statement

Santos. Comparing Laparoscopic Elective Sigmoid Resection With Conservative Treatment in Improving Quality of Life of Patients With Diverticulitis. *JAMA Surg*. Published November 18, 2020.
10.1001/jamasurg.2020.5151

Data
Data available: No

Additional Information
Explanation for why data not available: Study permissions do not permit sharing individual patient data
